# Supplementary material for: Bio-inspired nano-traps for uranium extraction from seawater and recovery from nuclear waste
Source: Nat Commun. 2018 Apr 24;9:1644. doi: 10.1038/s41467-018-04032-y (PMC5915388; doi:10.1038/s41467-018-04032-y)
Supplement: Supplementary file 1 — Supplementary Information [file 41467_2018_4032_MOESM1_ESM.pdf]

Supplementary Information

Bio-Inspired Nano-Traps for Uranium Extraction from Seawater and Recovery from Nuclear Waste

Sun et al.

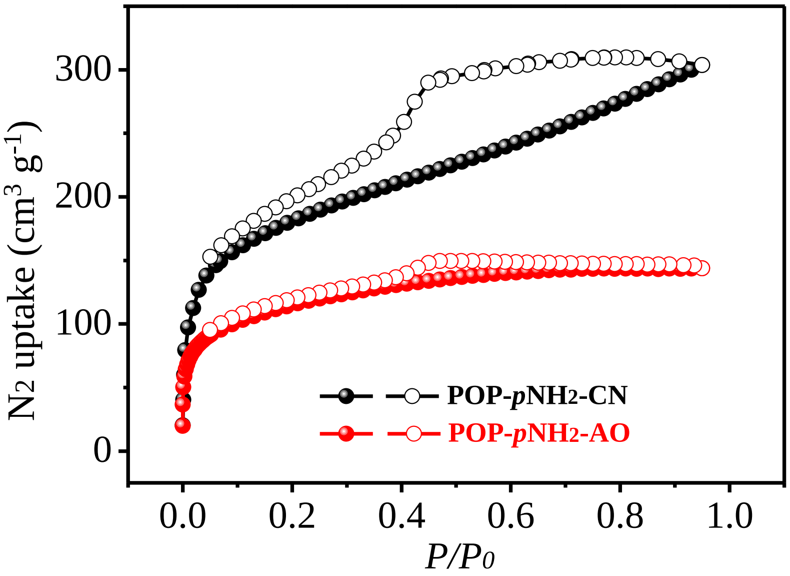

Supplementary Figure 1 | N_2 sorption isotherms collected at 77 K. The BET surface area of POP-*p*NH_2-CN and POP-*p*NH_2-AO were calculated to be 631 cm^3 g^{-1} and 397 cm^3 g^{-1}, respectively.

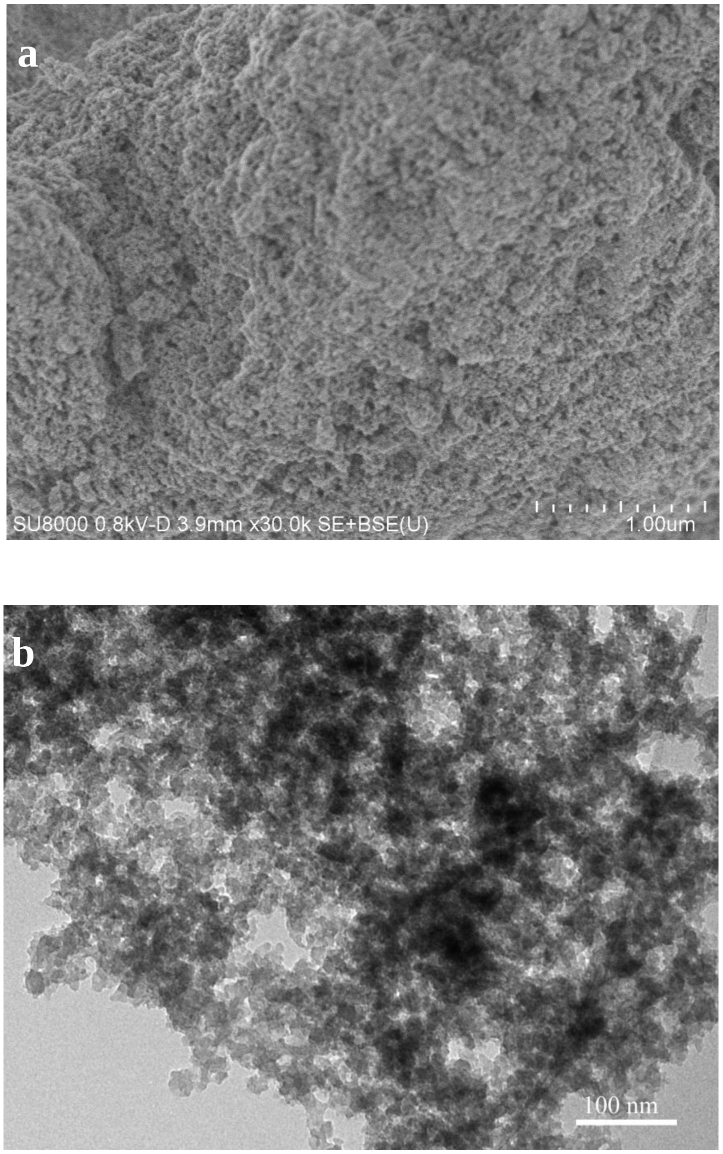

Supplementary Figure 2 | SEM (a) and TEM (b) images of POP-*p*NH_2-AO.

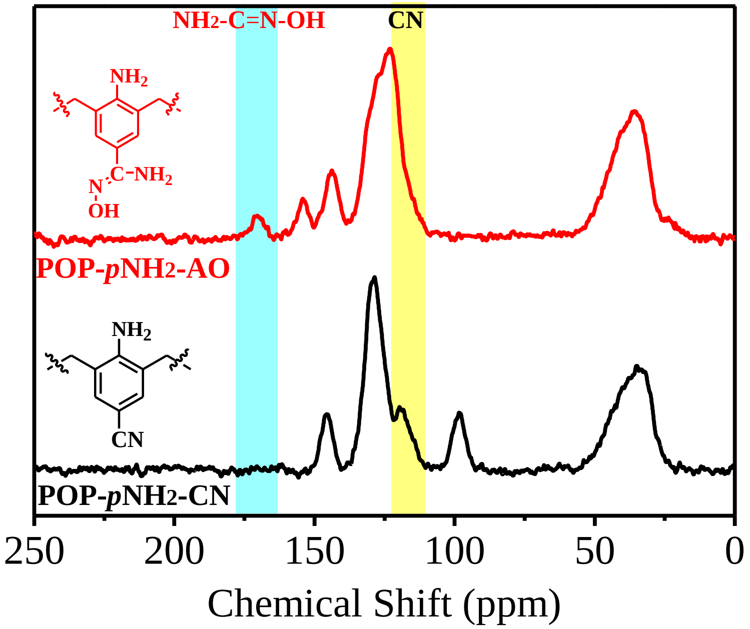

Supplementary Figure 3 | ^{13}C CP/MAS NMR spectra. The successful transformation from nitrile group into amidoxime group is verified by the disappearance of the peak at 119.7 ppm, which is related to CN groups and the concomitant emergence of the peak at 170.3 ppm, which is attributed to the amidoxime groups.

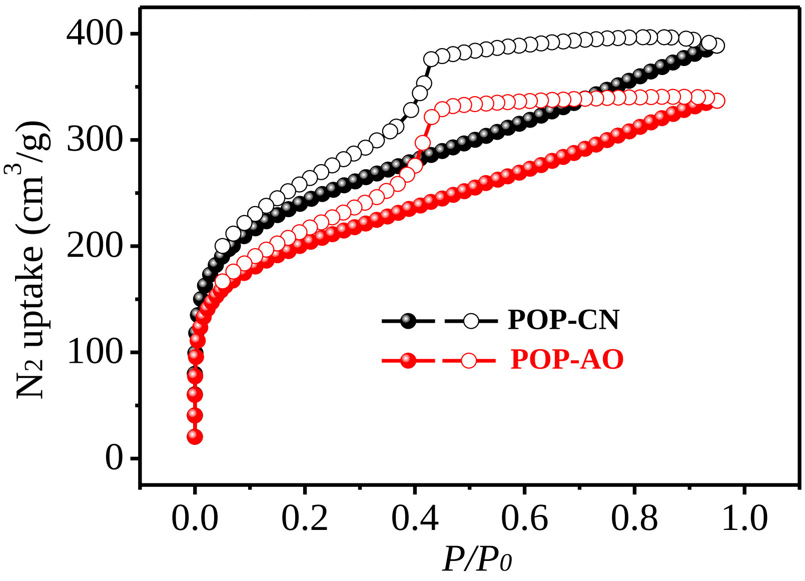

Supplementary Figure 4 | N_2 sorption isotherms collected at 77 K. The BET surface area of POP-CN and POP-AO were calculated to be 834 $cm^3\ g^{-1}$ and 696 $cm^3\ g^{-1}$, respectively.

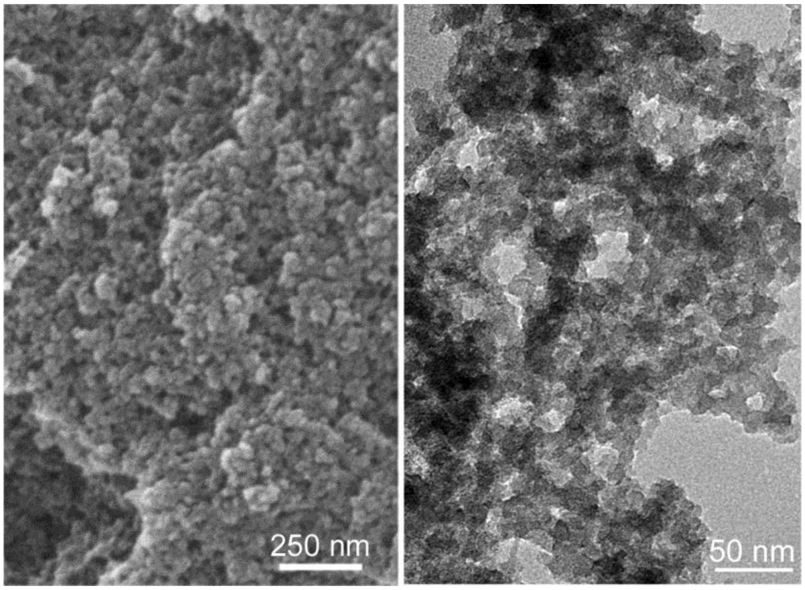

Supplementary Figure 5 | SEM (left) and TEM (right) images of POP-AO.

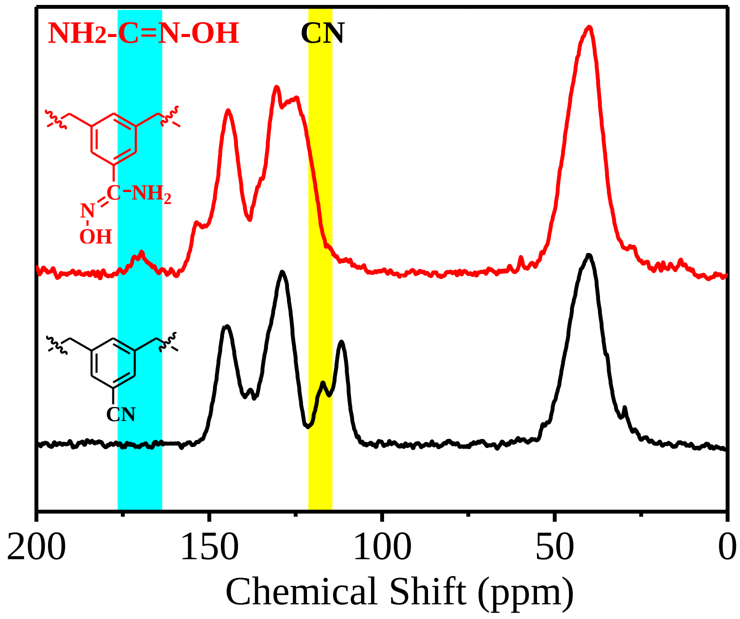

Supplementary Figure 6 | ^{13}C CP/MAS NMR spectra. Successful transformation of the nitrile group into the amidoxime group is verified by the disappearance of the peak at 117.4 ppm, which is related to the nitrile groups and the concomitant emergence of the peak at 170.3 ppm, which is attributed to the amidoxime groups.

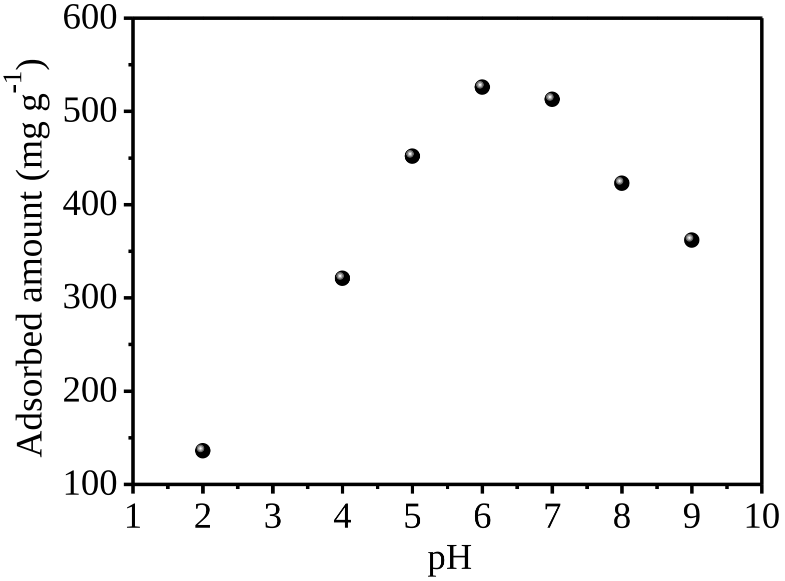

Supplementary Figure 7 | The pH dependence of POP-*o*NH$_2$-AO (3 mg) uptake capacities with uranium concentration 7.56 ppm (400 ppm) and 3 h contact time.

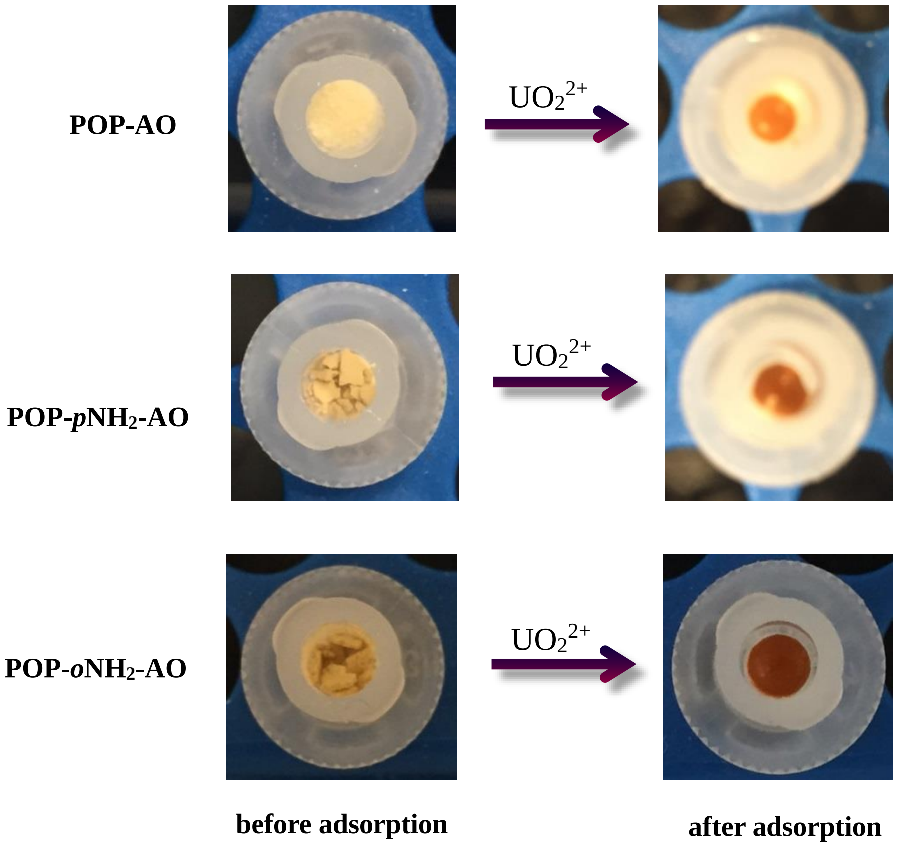

Supplementary Figure 8 | Photos of various adsorbent materials before and after uranium inclusion.

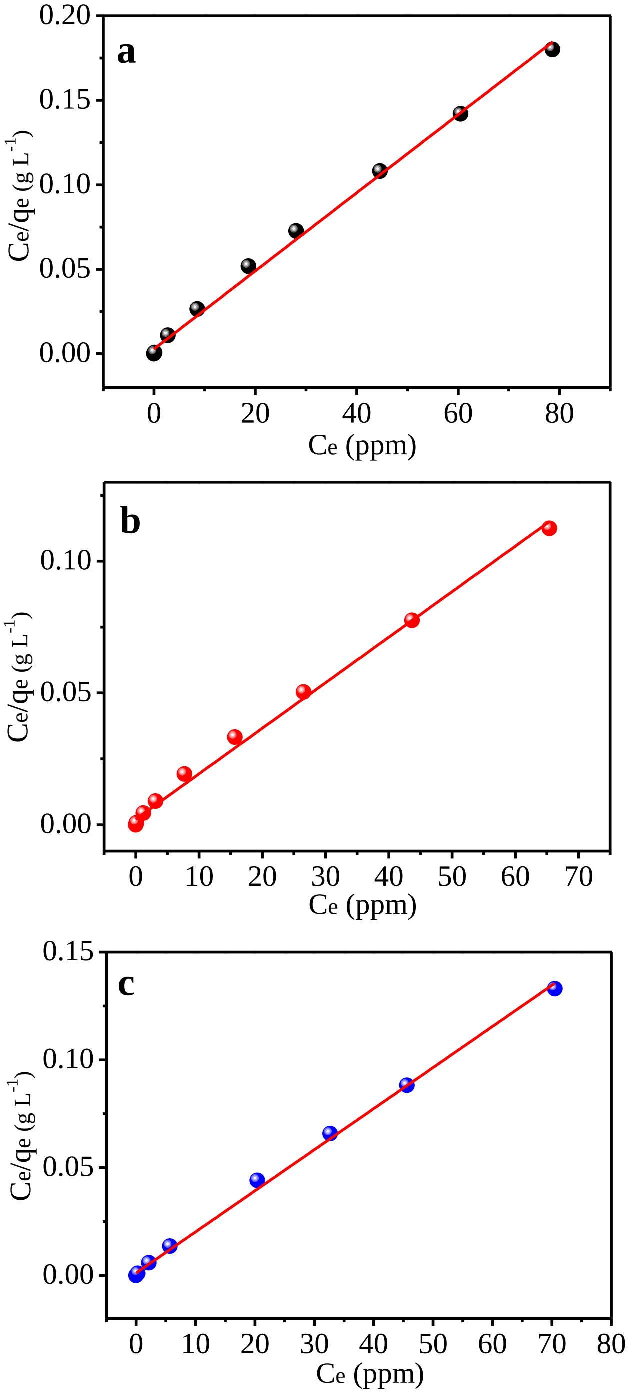

Supplementary Figure 9 | Linear regression by fitting the equilibrium data with the Langmuir adsorption model shown in Fig. 2a of the main text. (a) POP-AO, (b) POP-*p*NH_2-AO, and (c) POP-*o*NH_2-AO, respectively.

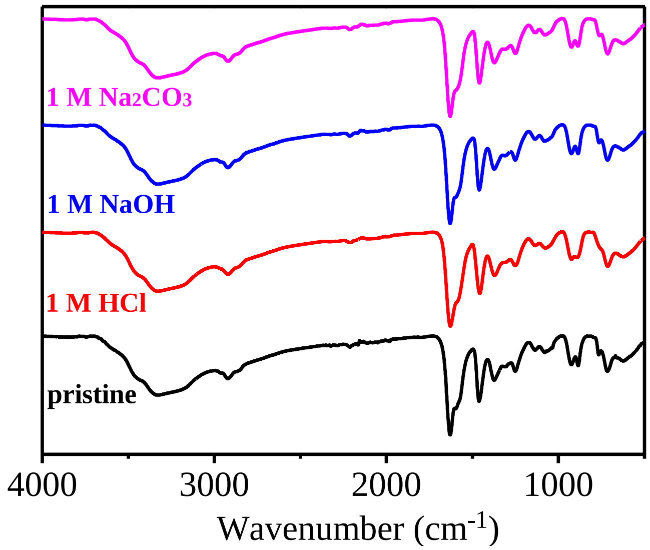

Supplementary Figure 10 | IR spectra of POP-*o*NH$_2$-AO before and after treatment with various conditions. IR spectra of COF-TpDb-AO before and after treatment for 24 h in 1 M acid and base aqueous solutions. Negligible change in IR spectra was observed after exposure to the above conditions, indicating its chemical stability.

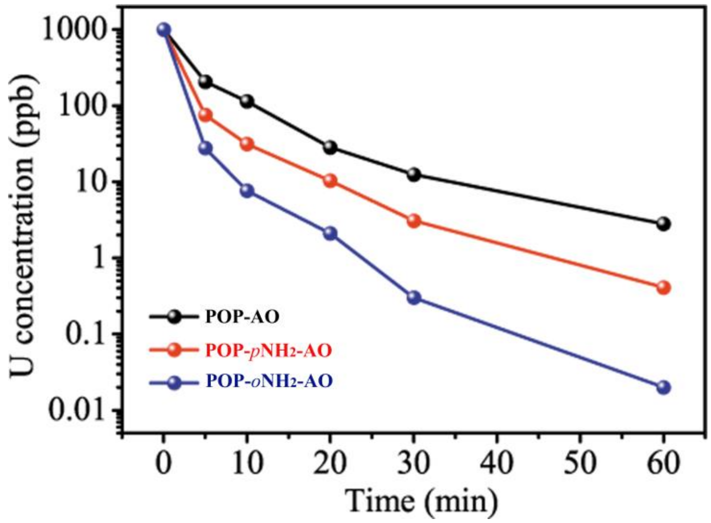

Supplementary Figure 11 | Uranium removal efficiency from real world water. The kinetics of uranium removal efficiency of POP-AO, POP-*p*NH_2-AO, and POP-*o*NH_2-AO from potable water spiked with uranium (1000 ppb) at V:m = 50000 mL g^{-1}.

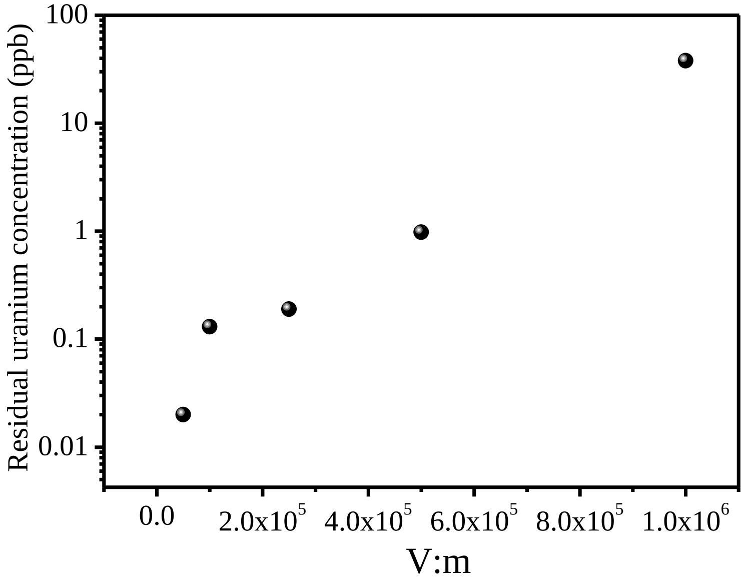

Supplementary Figure 12 | The effect of the ratio of solution volume (V) to POP-*o*NH_2-AO mass (m) on the residual concentration of uranium. Potable water spiked with uranium (1000 ppb) and 3 h contact time.

To test the ability of the adopted computational method to reproduce X-ray crystallographic data for $UO_2(AO)_2$ and $UO_2(o\text{NH}_2\text{-AO})_2$ complexes, we have performed geometry optimizations of $UO_2(AO)_2$ and $UO_2(o\text{NH}_2\text{-AO})_2$ at the M06/SSC/6-311++G(d,p) level of theory. As seen from Supplementary Fig. 13 and Supplementary Table 4, the calculations agree well with experiment, justifying the choice of the DFT functional and corresponding basis set.

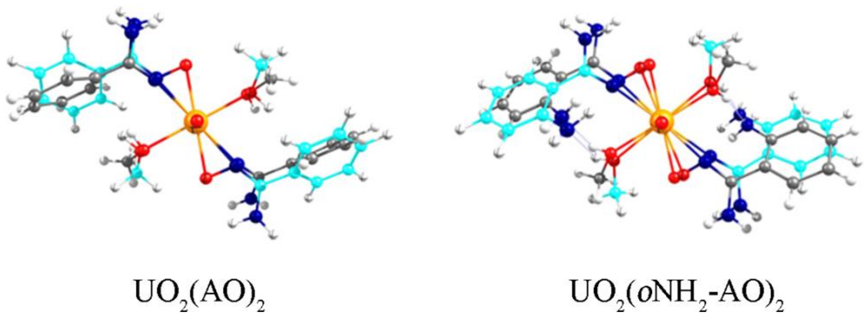

Supplementary Figure 13 | Overlays of the DFT optimized (turquoise carbon backbone) and experimental X-ray complex geometries (grey carbon backbone).

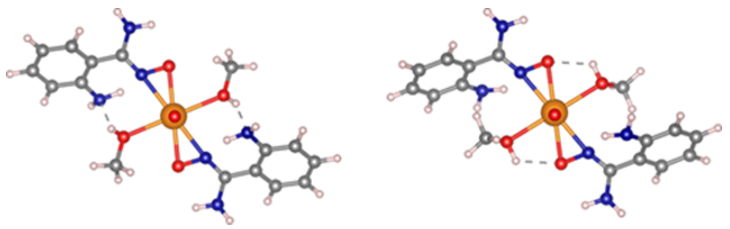

0.00 **$UO_2(oNH_2\text{-}AO)_2$** **H_2N - - $HOCH_3$**

4.73 **$UO_2(oNH_2\text{-}AO)_2$** **=NO - - $HOCH_3$**

Supplementary Figure 14 | Assessing hydrogen bond strength in the 2-aminobenzamidoxime (*o*NH_2-AO) complex. Structure (left) having hydrogen bonds (H_2N - - $HOCH_3$) was found to be 4.73 kcal mol^{-1} more stable than structure (right) without H_2N - - $HOCH_3$ hydrogen bonding interactions. Level of theory: M06/SSC/6-311++G (d,p) and the SMD solvation model.

DFT calculations at the M06/SSC/6-311++G** level of theory were performed to elucidate the optimal coordination modes and geometries of $UO_2(o NH_2\text{-}AO)^+$ and $UO_2(o NH_2\text{-}AO)_2$ in an aqueous environment. In these calculations we adopted a mixed cluster-continuum model, where the first coordination shell of the complexes was treated explicitly (water molecules), while the bulk solvent effects were represented by the SMD solvation model[13]. The most stable structures of the complexes are shown below. Consistent with the single-crystal X-ray diffraction data (see above), our calculations show that the *o*NH_2-AO ligands are bound to the uranyl cation in η^2 fashion through oxygen and nitrogen atom, while the amino groups at the *ortho* position form hydrogen bonds with adjacent water molecules.

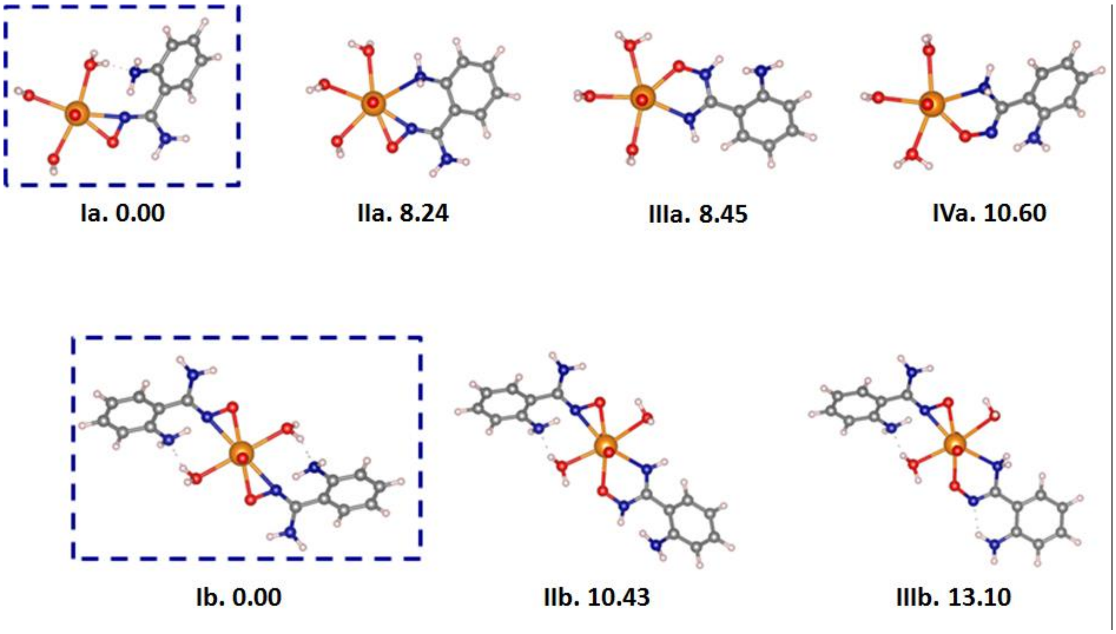

Supplementary Figure 15 | $UO_2(o NH_2\text{-}AO)_n^{2-n}$ (n = 1-2) structures and their relative energies in aqueous solution (kcal mol^{-1}). Level of theory: M06/SSC/6-311++G (d,p) and the SMD solvation model.

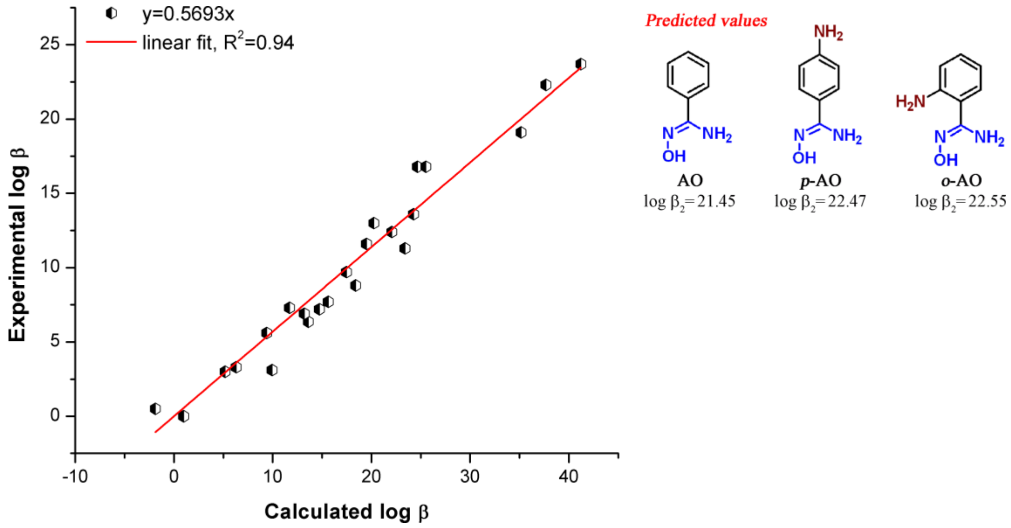

Supplementary Figure 16 |Assessing stability constants for the amine-substituted benzamidoxime ligands. The *o*NH_2-AO ligand shows strong complexation with the uranyl species, which is exemplified by high stability constants (log β_1 = 12.94; log β_2 = 22.55) in aqueous solution. The analogous stability constants for the *p*NH_2-AO and AO ligands were calculated to be lower (log β_1 = 13.35; log β_2 = 22.47 and log β_1 = 12.56; log β_2 = 21.45, respectively), suggesting that the amino group and hydrogen bonding interactions play an important role in the complexation process. Correlation scheme above and the corresponding computational protocols for predicting log β values of uranyl complexes were established in the previous studies [for details see: Vukovic, S. et al. Predicting stability constants for uranyl complexes using density functional theory. *Inorg. Chem.* **54**, 3995-4001, (2015); Mehio, N. et al. Quantifying the binding strength of salicylaldoxime-uranyl complexes relative to competing salicylaldoxime-transition metal ion complexes in aqueous solution: a combined experimental and computational study. *Dalton Trans.* 45, 9051-9064, (2016)].

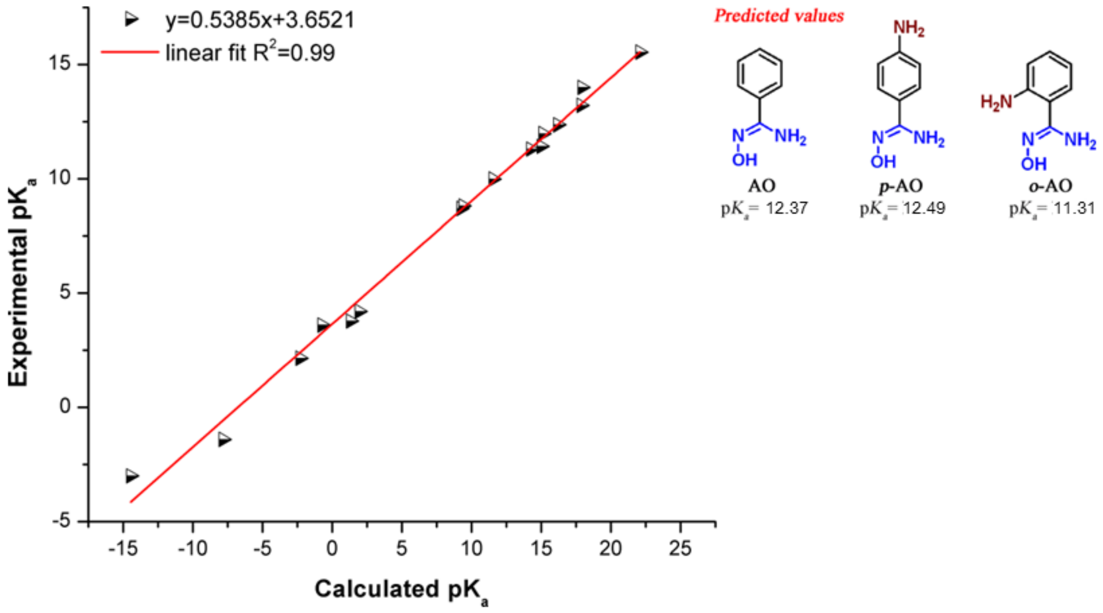

Supplementary Figure 17 | Assessing pK_a for the amine-substituted benzamidoxime ligands. *o*NH_2-AO possesses the lowest pK_a value among amidoxime-type ligands, meaning that it would require less energy for deprotonating to bind the UO_2^{2+} ion as compared to the *p*NH_2-AO and AO ligands. This property can be considered as another advantage of *o*NH_2-AO over *p*NH_2-AO and AO. Correlation scheme above and the corresponding computational protocols for predicting pK_a values of uranyl complexes were established in the previous study (for details see: Mehio, N., et al. Acidity of the amidoxime functional group in aqueous solution: a combined experimental and computational study. *J. Phys. Chem. B* **119**, 3567-3576, (2015).

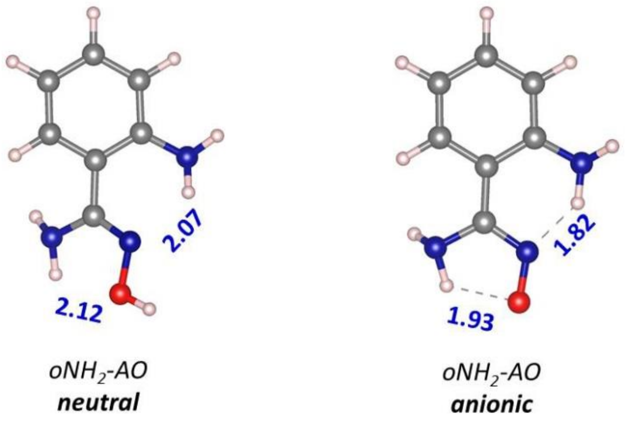

Supplementary Figure 18 | Strong intramolecular hydrogen bonds in the anionic form of *o*NH_2-AO.

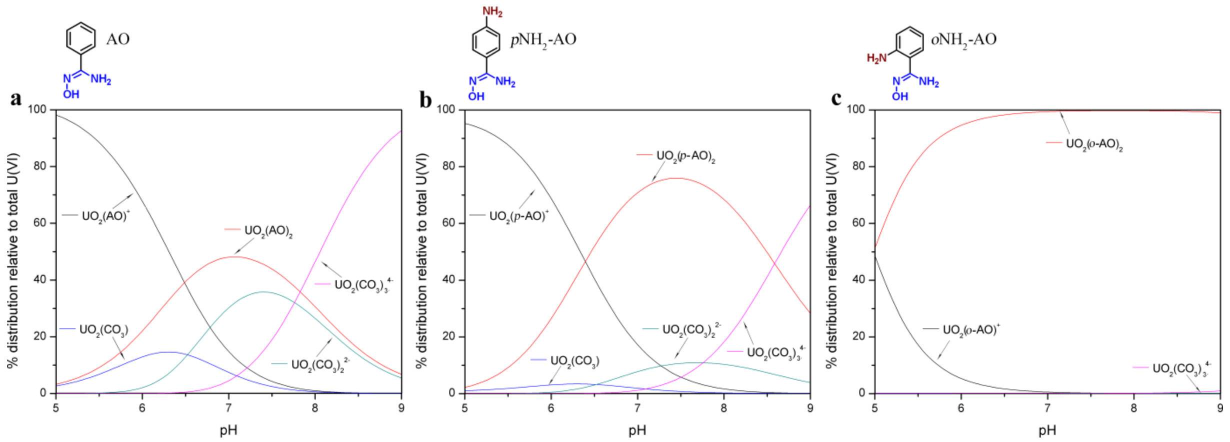

Supplementary Figure 19 | Simulating speciation diagrams. The simulated speciation diagrams for different functionalities: (a) benzamidoxime (AO), (b) 4-aminobenzamidoxime (*p*NH_2-AO), and (c) 2-aminobenzamidoxime (*o*NH_2-AO) demonstrate superior performance of *o*NH_2-AO followed by *p*NH_2-AO and AO at seawater simulant conditions. Atomic weight of uranium was used to convert 10.3 ppm of uranium to molar concentration (4.38×10^{-5} M). Concentrations used in the simulations: [AO] = 0.001 M; [*p*NH_2-AO] = 0.001 M; [*o*NH_2-AO] = 0.001 M; [UO_2^{2+}] = 4.38×10^{-5} M; [CO_3^{2-}] = 0.0023 M.

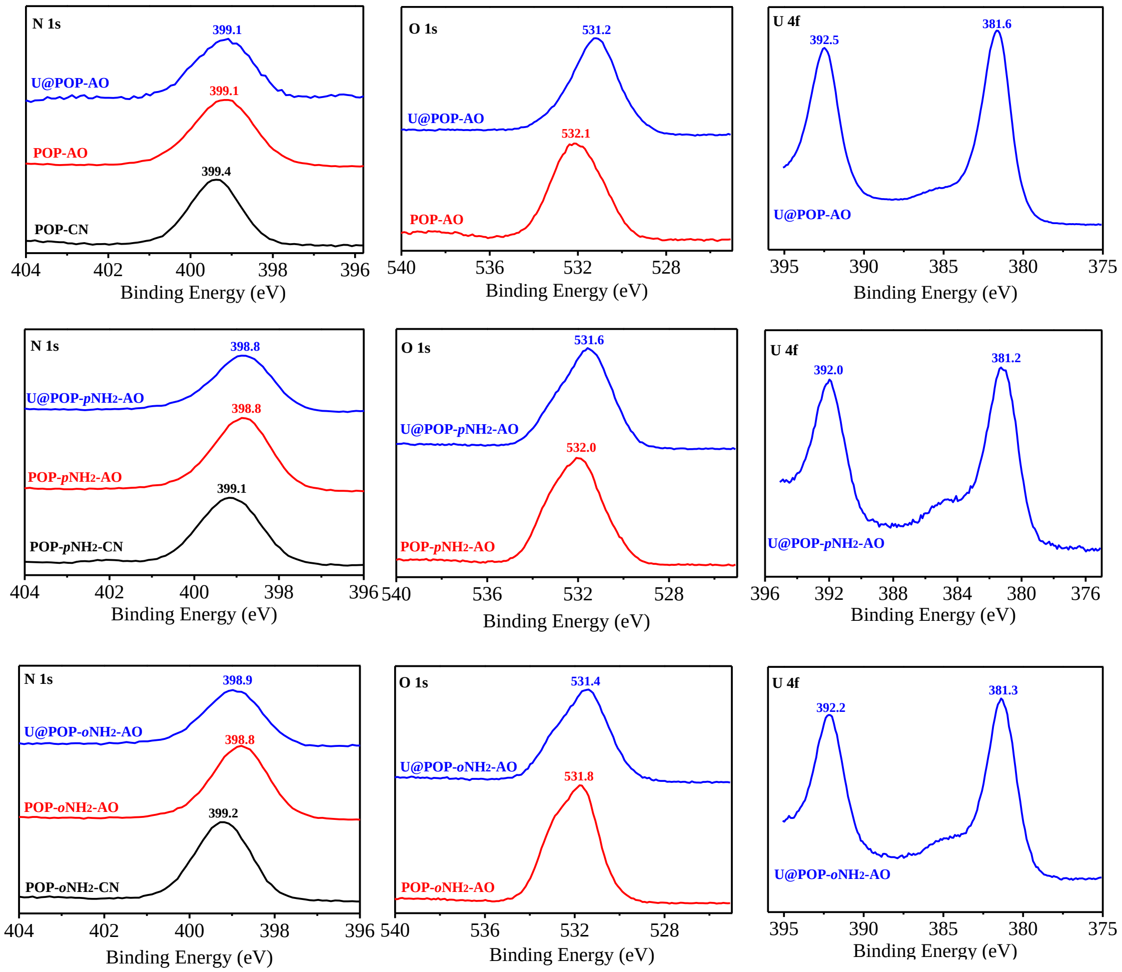

Supplementary Figure 20 | N1s, O1s, and U4f XPS spectra.

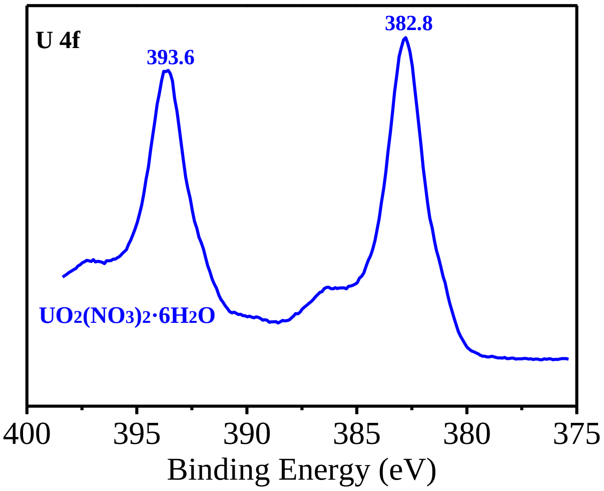

Supplementary Figure 21 | U4f XPS spectrum of $UO_2(NO_3)_2{\cdot}6H_2O$.

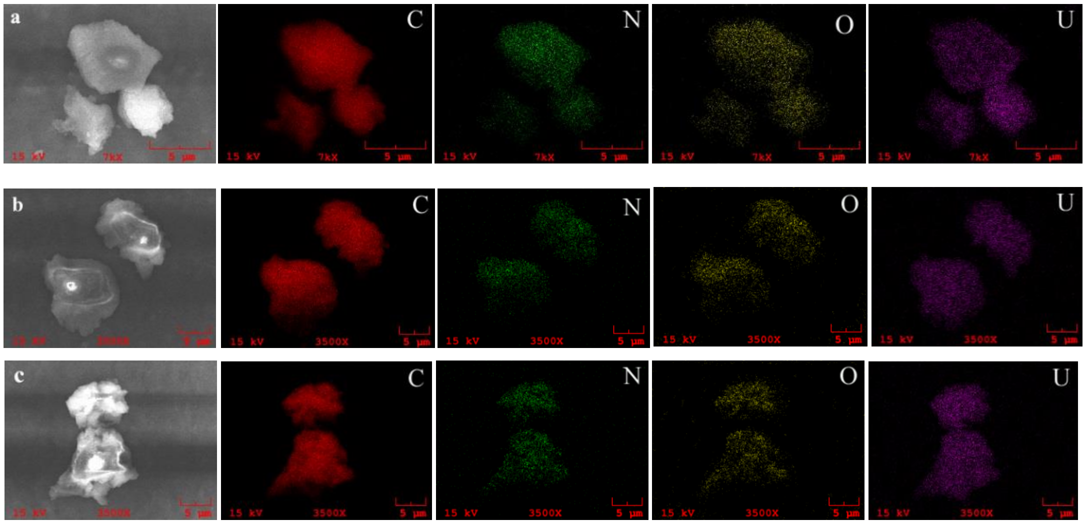

Supplementary Figure 22 | SEM images and corresponding EDX mapping. (a) U@POP-AO, (b) U@POP-*p*NH_2-AO, and (c) U@POP-*o*NH_2-AO.

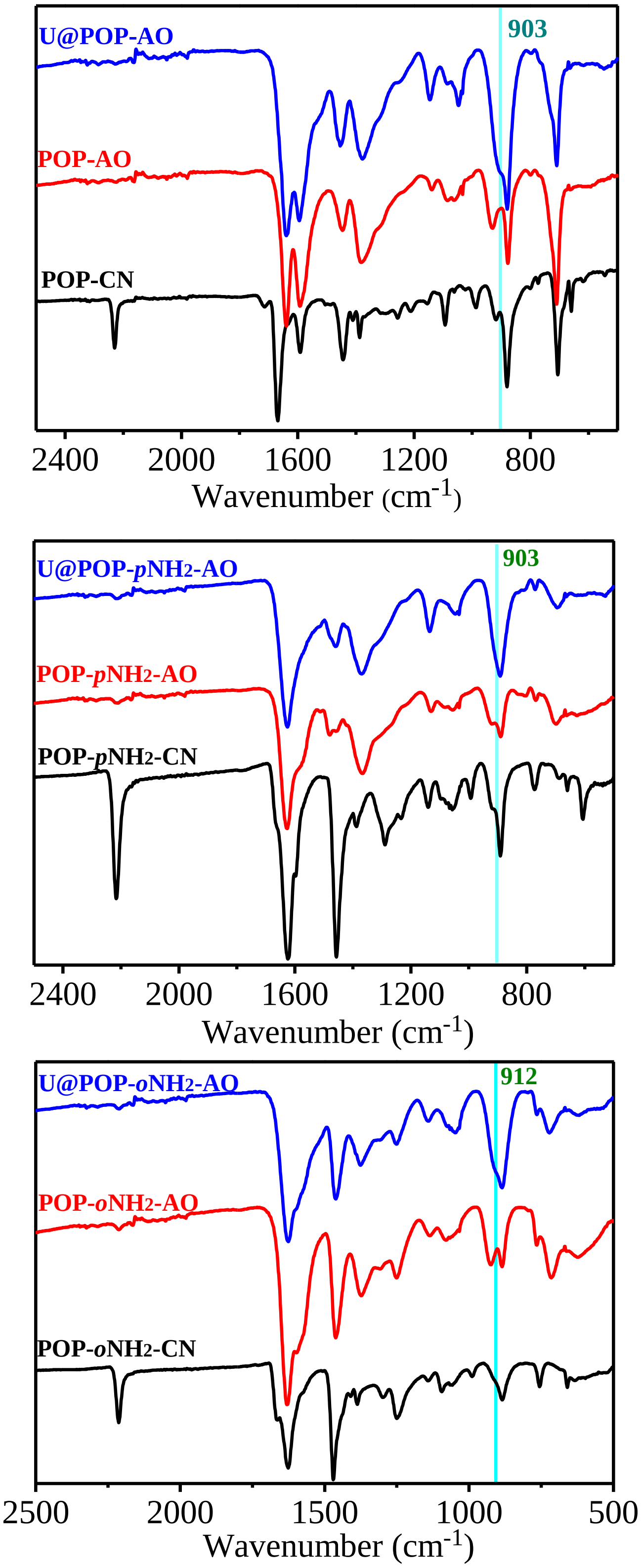

Supplementary Figure 23 | IR spectra.

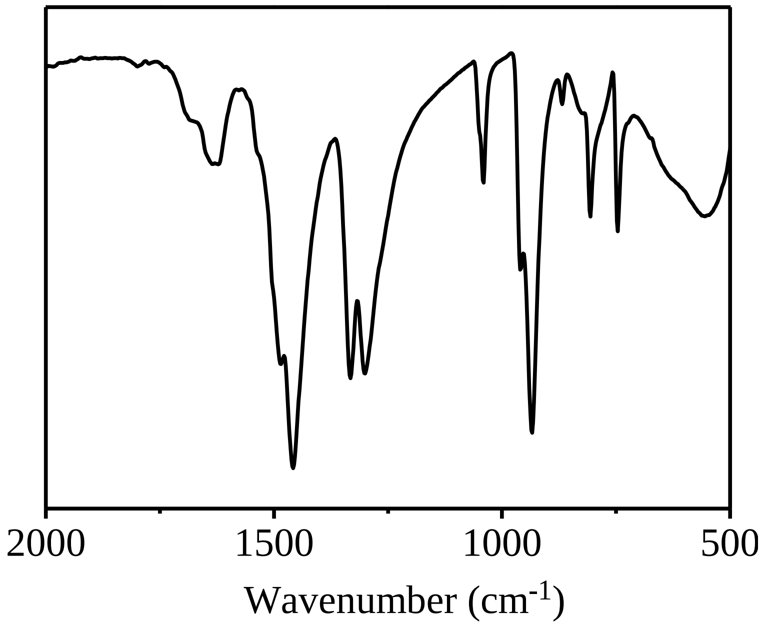

Supplementary Figure 24 | IR spectrum of $UO_2(NO_3)_2{\cdot}6H_2O$.

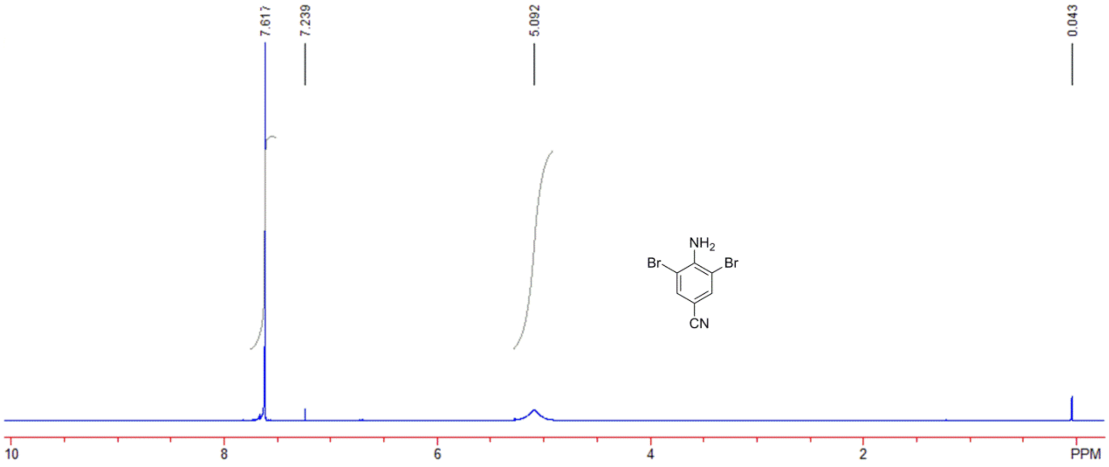
7.617
7.239
5.092
0.043
NH2
Br
Br
CN
10
8
6
4
2
PPM

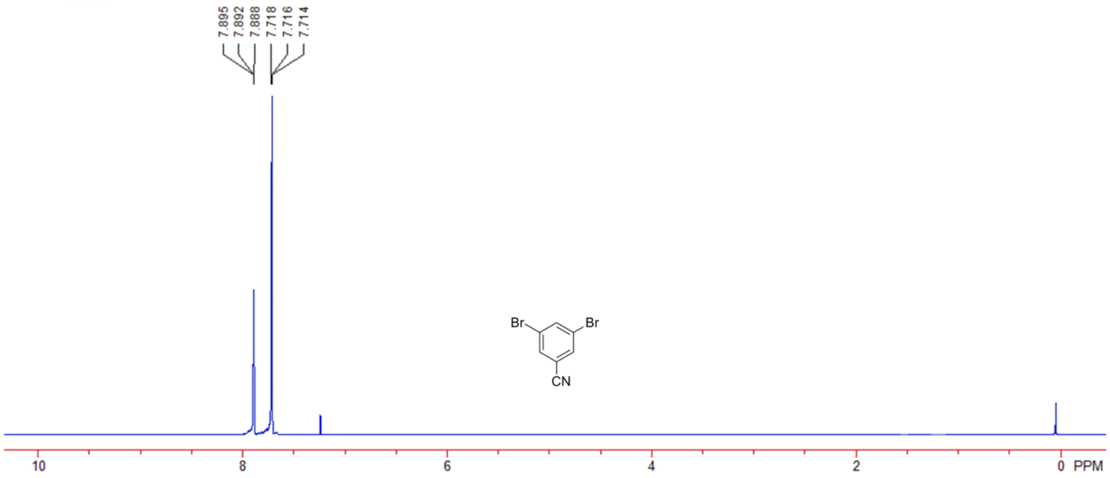
7.895
7.892
7.888
7.718
7.716
7.714
Br
Br
CN
10
8
6
4
2
0 PPM

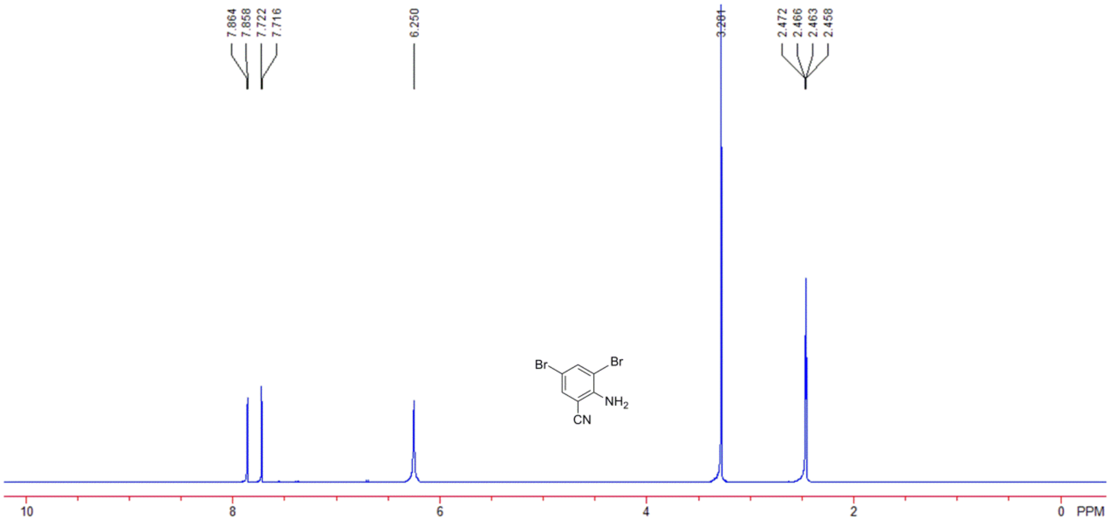
7.864
7.858
7.722
7.716
6.250
3.281
2.472
2.466
2.463
2.458
Br
Br
NH2
CN
10
8
6
4
2
0 PPM

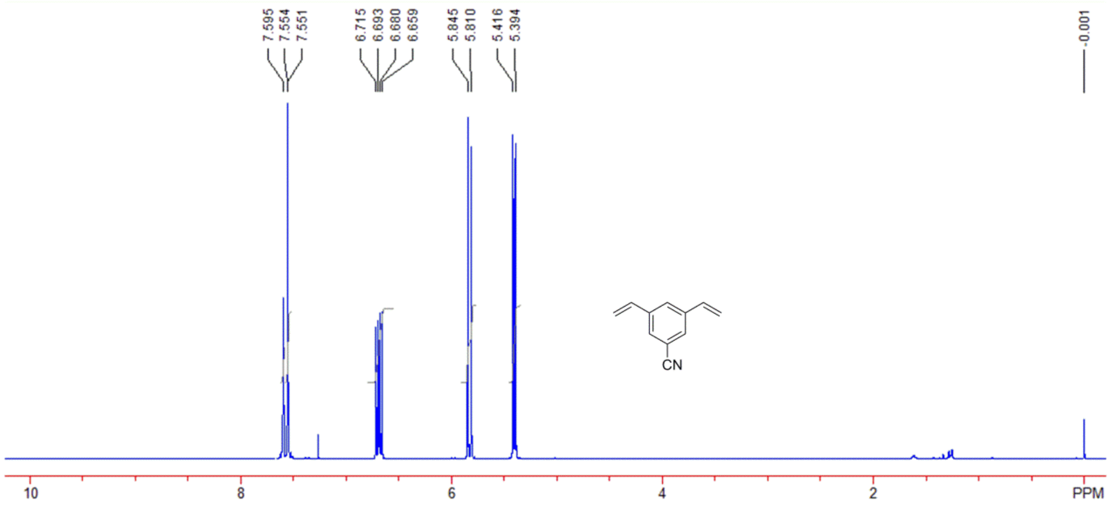
7.595
7.554
7.551
6.715
6.693
6.680
6.659
5.845
5.810
5.416
5.394
-0.001
CN
10
8
6
4
2
PPM

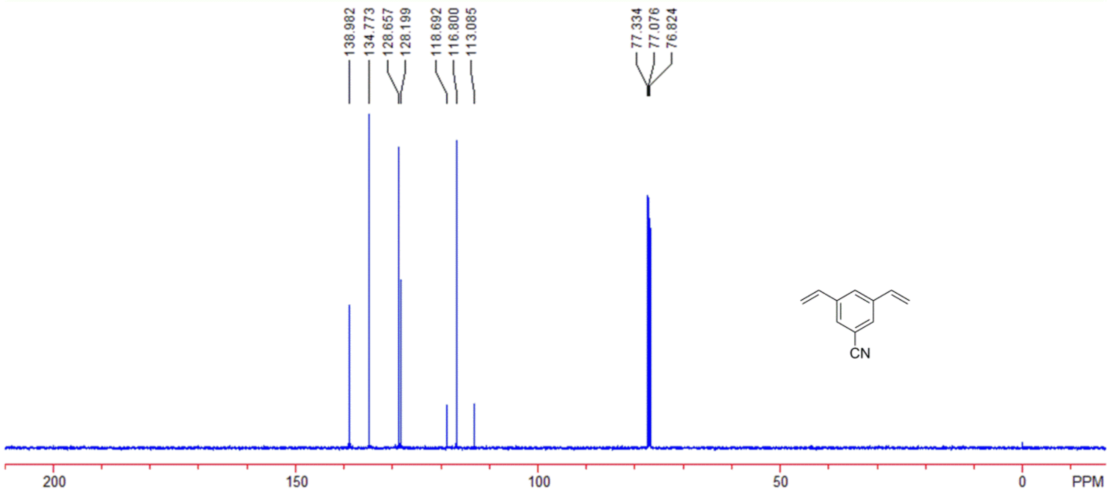
138.982
134.773
128.657
128.199
118.692
116.800
113.085
77.334
77.076
76.824
CN
200
150
100
50
0
PPM

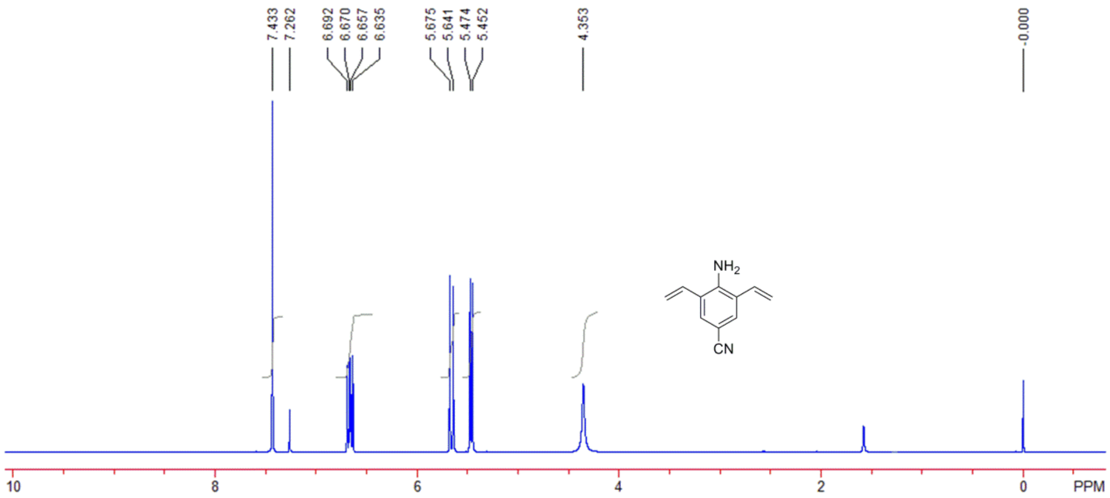
7.433
7.262
6.692
6.670
6.657
6.635
5.675
5.641
5.474
5.452
4.353
-0.000
NH2
CN
10
8
6
4
2
0
PPM

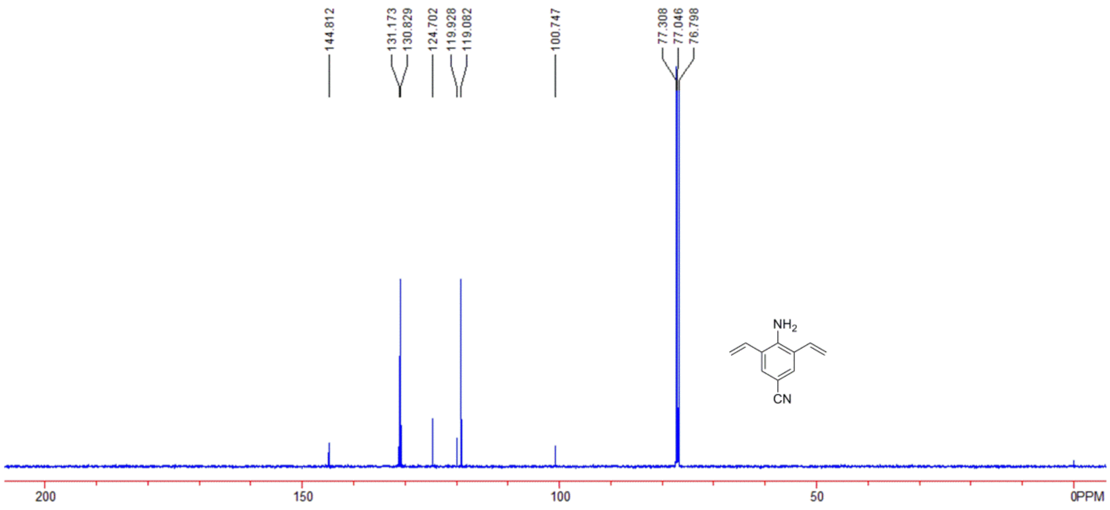

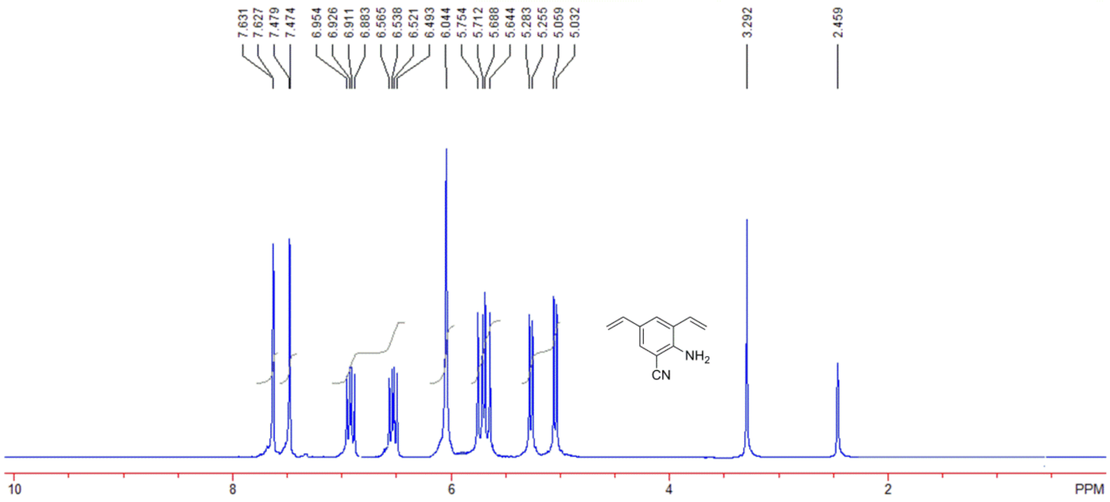

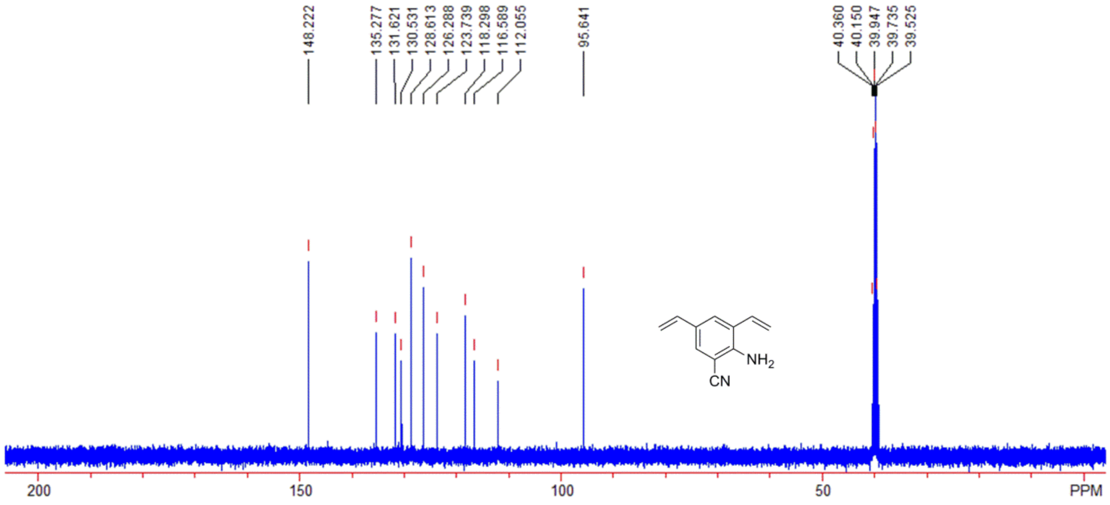

Supplementary Figure 25 | ^{1}H NMR spectra of various compounds.

Supplementary Tables

Supplementary Table 1 | Uranium sorption performance of representative adsorbents in the literature.

Adsorbents	Water (mg g^{-1})	Artificial seawater (mg g^{-1})	Seawater (mg g^{-1})
MSPh-III (phosphonic acid-modified mesoporous material)[a]	182	66.7	--
Mesoporous Carbon Materials[b]	97	67	--
PAO/PVDF[c]	--	1.6	--
S-CP40*E-AO[d]	--	57	--
Am-p(AN-c-MAc)[e]	51.5		--
MOF-76[f]	298		--
S_x-LDH[g]	330		--
MIL-101-DETA[h]	350		--
(MIL-101(Cr)-triazole-COOH[i]	304		--
V_2CT_x[j]	174	377	--
ND-AO[k]	212	121	--
Am-p(AN-c-MAc) particles[l]	51.5	--	--
KIT-6-80-P[m]	56		--
AF series adsorbents[n]		200	--
F-AA2 fiber[o]		50	--
FJSM-SnS[p]	338	--	--
S_x-LDH[q]	330	--	0.00072
zero-valent iron[r]	2400	(50-300 ppm) 240-1410	--
$K_2MnSn_2S_6$ (KMS-1)[s]	382	--	0.00029
p(2DVB-VBC)-2PAN[t]	--	80	1.99
**This work (POP-*o*NH_2-AO)**	**530**	**290**	**4.36**

[a] Vivero-Escoto, J. L.; Carboni, M.; Abney, C. W.; deKrafft, K. E. & Lin, W. Organo-functionalized mesoporous silicas for efficient uranium extraction. *Micropor. Mesopor. Mater.* **180**, 22-31 (2013).

[b] Carboni, M.; Abney, C. W.; Taylor-Pashow, K. M. L.; Vivero-Escoto, J. L. & Lin, W. Uranium sorption with functionalized mesoporous carbon materials. *Ind. Eng. Chem. Res.* **52**, 15187-15197 (2013).

[c] Xie, S.; Liu, X.; Zhang, B.; Ma, H.; Ling, C.; Yu, M.; Li, L. & Li, J. Electrospun nanofibrous adsorbents for uranium extraction from seawater. *J. Mater. Chem. A* **3**, 2552-2558 (2015).

[d] Gunathilake, C.; Górka, J.; Dai, S. & Jaroniec, M. Amidoxime-modified mesoporous silica for uranium adsorption under seawater conditions. *J. Mater. Chem. A* **3**, 11650-11659 (2015).

[e] Sahiner, N.; Yu, H.; Tan, G.; He, J.; John, V. T. & Blake, D. A. *ACS Appl. Mater. Interfaces* **4**, 163-170 (2012).
[f] Yang, W.; Bai, Z.-Q.; Shi, W.-Q.; Yuan, L.-Y.; Tian, T.; Chai, Z.-F.; Wang, H. & Sun, Z.-M. MOF-76: from a luminescent probe to highly efficient U^{VI} sorption material. *Chem. Commun.* **49**, 10415-10417 (2013).
[g] Ma, S.; Huang, L.; Ma, L.; Shim, Y.; Islam, S. M.; Wang, P.; Zhao, L.-D.; Wang, S.; Sun, G.; Yang, X. & Kanztzidis, M. G. Efficient uranium capture by polysulfide/layered double hydroxide composites. *J. Am. Chem. Soc.* **13**, 3670-3677 (2015).
[h] Bai, Z.-Q.; Yuan, L.-Y.; Zhu, L.; Liu, Z.-R.; Chu, S.-Q.; Zheng, L.-R.; Zhang, J.; Chai, Z.-F. & Shi, W.-Q. Introduction of amino groups into acid-resistant MOFs for enhanced U(VI) sorption. *J. Mater. Chem. A* **3**, 525-534 (2015).
[i] Li, L.; Ma, W.; Shen, S.; Huang, H.; Bai, Y. & Liu, H. A combined experimental and theoretical study on the extraction of uranium by amino-derived metal-organic frameworks through post-synthetic strategy. *ACS Appl. Mater. Interfaces* **8**, 31032-31041 (2016).
[j] Wang, L.; Yuan, L.; Chen, K.; Zhang, Y.; Deng, Q.; Du, S.; Huang, Q.; Zheng, L.; Zhang, J.; Chai, Z.; Barsoum, M. W.; Wang, X. & Shi, W. Loading actinides in multilayered structures for nuclear waste treatment: the first case study of uranium capture with vanadium carbide MXene. *ACS Appl. Mater. Interfaces* **8**, 16396-16403 (2016).
[k] Li, Y.; Wang, L.; Li, B.; Zhang, M.; Wen, R.; Guo, X.; Li, X.; Zhang, J. & Li, S. Pore-Free matrix with cooperative chelating of hyperbranched ligands for high-performance separation of uranium. *ACS Appl. Mater. Interfaces* **8**, 28853-28861 (2016).
[l] Sahiner, N.; Yu, H.; Tan, G.; He, J.; John, V. T. & Blake, D. A. Highly porous acrylonitrile-based submicron particles for UO_2^{2+} absorption in an immunosensor assay. *ACS Appl. Mater. Interfaces* **4**, 163-170 (2012).
[m] Lebed, P. J.; Savoie, J.-D.; Florek, J.; Bilodeau, F.; Larivière, D. & Kleitz, F. Large pore mesostructured organosilica-phosphonate hybrids as highly efficient and regenerable sorbents for uranium sequestration. *Chem. Mater.* **24**, 4166-4176 (2012).
[n] Das, S.; Oyola, Y.; Mayes, R. T.; Janke, C. J.; Kuo, L.-J.; Gill. G.; Wood, J. R. & Dai, S. Extracting uranium from seawater: promising AF series adsorbents. *Int. Eng. Chem. Res.* **55**, 4110-4117 (2015).
[o] Chatterjee, S.; Bryantsev, V. S.; Brown, S.; Johnson, J. C.; Grant, C. D.; Matyes, R. T.; Hay, B. P.; Dai, S. & Saito, T. Synthesis of naphthalimidedioxime ligand-containing fibers for uranium adsorption from seawater. *Int. Eng. Chem. Res.* **55**, 4161-4169 (2015).
[p] Feng, M.-L.; Sarma, D.; Qi, X.-H.; Du, K.-Z.; Huang, X.-Y. & Kanatzidis, M. G. Efficient removal and recovery of uranium by a layered organic-inorganic hybrid thiostannate. *J. Am. Chem. Soc.* **138**, 12578-12585 (2016).
[q] Ma, S.; Huang, L.; Ma, L.; Shim, Y.; Islam, S. M.; Wang, P.; Zhao, L.-D.; Wang, S.; Sun, G.; Yang, X. & Kanatzidis, M. G. Efficient uranium capture by polysulfide/layered double hydroxide composites. *J. Am. Chem. Soc.* **137**, 3670-3677 (2015).
[r] Ling, L. & Zhang, W.-x. Enrichment and encapsulation of uranium with iron nanoparticle. *J. Am. Chem. Soc.* **137**, 2788-2791 (2015).
[s] Manos, M. J. & Kanatzidis, M. G. Layered metal sulfides capture uranium from seawater. *J. Am. Chem. Soc.* **134**, 16441-16446 (2012).
[t] Yue, Y.; Mayes, R. T.; Kim, J.; Fulvio, P. F.; Sun, X.-G.; Tsouris, C.; Chen, J.; Brown, S. & Dai, S. Seawater uranium sorbents: preparation from a mesoporous copolymer initiator by atom-transfer radical polymerization. *Angew. Chem. Int. Ed.* **52**, 13458-13462 (2013).

Supplementary Table 2 | Crystal data and structure refinement for UO_2(*o*NH_2-AO)$_2$(MeOH)$_2$.

Identification code	CCDC 1547954
Empirical formula	$C_{20}H_{40}N_6O_{10}U$
Moiety formula	$[UO_2(C_7H_8N_3O)_2(CH_3OH)_2]\cdot(CH_3OH)_4$
Temperature/K	100.0
Crystal system	monoclinic
Space group	*C*2/*c*
a/Å	22.6512(6)
b/Å	6.7491(2)
c/Å	20.7330(5)
α/°	90
β/°	118.7730(10)
γ/°	90
Volume/Å^3	2778.23(13)
Z	4
ρ_{calc}g/cm^3	1.823
μ/mm^{-1}	16.975
F(000)	1496.0
Crystal size/mm^3	0.08 × 0.03 × 0.02
Radiation	CuKα (λ = 1.54178)
2Θ range for data collection/°	8.908 to 136.472
Index ranges	$-27 \leq h \leq 27$, $-8 \leq k \leq 8$, $-22 \leq l \leq 24$
Reflections collected	12939
Independent reflections	2541 [R_{int} = 0.0580, R_{sigma} = 0.0373]
Data/restraints/parameters	2541/29/209
Goodness-of-fit on F^2	1.095
Final R indexes [I>=2σ (I)]	R_1 = 0.0246, wR_2 = 0.0465
Final R indexes [all data]	R_1 = 0.0458, wR_2 = 0.0517
Largest diff. peak/hole / e Å^{-3}	0.87/-0.45

Supplementary Table 3 | Geometric Parameters of X-ray Structures for (A) $[UO_2(AO)_2(MeOH)_2]$ and (B) $[UO_2(oNH_2\text{-}AO)_2(MeOH)_2]$.

	A	B
**distances [Å]**		
U=O	1.796	1.784
U-N	2.438	2.417
U-O	2.352	2.448
O-N	1.422	1.412
C=N	1.293	1.304
U-OH	2.304	
**angles [deg]**		
U-O-N	76.1	75.3
U-N-O	69.5	70.3
O=U-O	87.9	87.3
O=U-N	92.8	89.3
O-N=C	114.2	115.8

Supplementary Table 4 | Selected bond lengths (Å) of the $UO_2(AO)_2$ and $UO_2(\textit{o}NH_2\text{-}AO)_2$ complexes calculated at the M06/SSC/6-311++G(d,p) level in comparison with the experimental $UO_2(AO)_2$ and $UO_2(\textit{o}NH_2\text{-}AO)_2$ crystallographic structures.

	U-O(oximate group)	U-N(oximate group)	U-O(methanol)
**X-ray diffraction**			
$UO_2(AO)_2$	2.36	2.44	2.42
**M06/SSC/6-311++G(d,p)**			
$UO_2(AO)_2$	2.36	2.44	2.57
**X-ray diffraction**			
$UO_2(\textit{o}NH_2\text{-}AO)_2$	2.35	2.41	2.45
**M06/SSC/6-311++G(d,p)**			
$UO_2(\textit{o}NH_2\text{-}AO)_2$	2.35	2.46	2.53

Supplementary Table 5 | Comparison of the strengths of ligand-uranyl interactions in complexes using natural bond orbital (NBO) method. Second-order stabilization energies ($E^{(2)}$, kcal/mol) suggest that ortho-amine-substituted ligand (*o*NH_2-AO) provides stronger donor-acceptor interactions with uranyl than para-amine-substituted (*p*NH_2-AO) and benzamidoxime (AO) ligands.

Complex	Donor NBO → Acceptor NBO in UO_2^{2+} complexes (kcal/mol)				Charge on UO_2^{2+} unit
	**LP_N→n^*_U (oximate group)**	**LP_O→n^*_U (oximate group)**	**LP_O→n^*_U (methanol)**	**Total**	
$UO_2(AO)_2(MeOH)_2$ 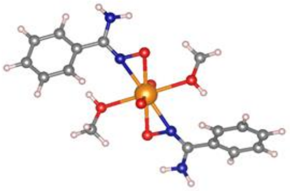	175.5	254.5	106.0	536.0	+0.52
UO_2(*p*NH_2-AO)$_2(MeOH)_2$ 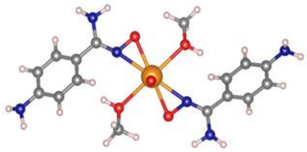	196.1	278.3	116.4	590.8	+0.51
UO_2(*o*NH_2-AO)$_2(MeOH)_2$ 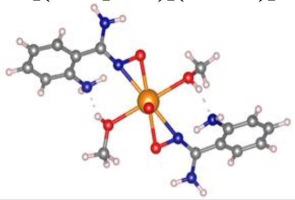	178.8	309.3	131.7	619.8	+0.49

Supplementary Table 6 | Simulating speciation diagrams. Equilibrium constants included in simulations for AO, *p*NH$_2$-AO, and *o*NH$_2$-AO; all at 25 °C and ionic strength (μ) = 0.

Aqueous species, reactions	log *β*
$H^+ + OH^- \leftrightarrows H_2O$	14.00[a]
$H^+ + CO_3^{2-} \leftrightarrows HCO_3^-$	10.33[b]
$2H^+ + CO_3^{2-} \leftrightarrows H_2CO_3$	16.68[b]
$H^+ + AO^- \leftrightarrows AOH$	12.37[c]
$H^+ + p$NH$_2$-AO $^- \leftrightarrows p$NH$_2$-AOH	12.49[c]
$H^+ + o$NH$_2$-AO $^- \leftrightarrows o$NH$_2$-AOH	11.31[c]
$UO_2^{2+} + AO^- \leftrightarrows UO_2(oNH_2$-AO$)^+$	12.56 [c]
UO_2^{2+} + 2AO $^- \leftrightarrows UO_2(oNH_2$-AO $)_2$	21.45[c]
$UO_2^{2+} + p$NH$_2$-AO$^- \leftrightarrows UO_2(pNH_2$-AO$)^+$	13.35[c]
$UO_2^{2+} + 2p$NH$_2$-AO$^- \leftrightarrows UO_2(pNH_2$-AO $)_2$	22.47[c]
$UO_2^{2+} + o$NH$_2$-AO$^- \leftrightarrows UO_2(oNH_2$-AO$)^+$	12.94[c]
$UO_2^{2+} + 2o$NH$_2$-AO$^- \leftrightarrows UO_2(oNH_2$-AO $)_2$	22.55[c]
$UO_2^{2+} + CO_3^{2-} \leftrightarrows UO_2(CO_3)$	9.94[d]
$UO_2^{2+} + 2CO_3^{2-} \leftrightarrows UO_2(CO_3)_2^{2-}$	16.61[d]
$UO_2^{2+} + 3CO_3^{2-} \leftrightarrows UO_2(CO_3)_3^{4-}$	21.84[d]
$UO_2^{2+} + OH^- \leftrightarrows UO_2(OH)^+$	5.25[e]
$UO_2^{2+} + 2OH^- \leftrightarrows UO_2(OH)_2$	12.15[e]
$UO_2^{2+} + 3OH^- \leftrightarrows UO_2(OH)_3^-$	20.25[e]
$UO_2^{2+} + 4OH^- \leftrightarrows UO_2(OH)_4^{2-}$	32.40[e]
$2UO_2^{2+} + OH^- \leftrightarrows (UO_2)_2(OH)^{3+}$	11.3[e]
$2UO_2^{2+} + 2OH^- \leftrightarrows (UO_2)_2(OH)_2^{2+}$	22.4[e]
$UO_2^{2+} + 2OH^- \leftrightarrows UO_2(OH)_2$ (s)	-22.0[a]

[a] Thuéry, P. & Nierlich, M. Crystal structure of a uranyl/p-tert-butylcalix[5]arene complex. *J. Inclusion Phenom. Mol. Recognit. Chem.* **27**, 13-20 (1997).

[b] Smith, R. M. & Martell, A. E. Critical stability constants. Plenum Press: New York (1981).

[c] This work. Values were predicted from correlations shown in Supplementary Figures 12-15.

[d] Ramamoorthy, S. & Santappa, M. Stability constants of some uranyl complexes. *Bull. Chem. Soc. Jpn.* **41**, 1330-1333 (1968).

[e] Grenthe, I.; Drozdzynski, J.; Fujino, T.; Buck, E. C.; Albrecht-Schmitt, T. E. & Wolf, S. F. *The chemistry of the actinide and transactinide elements*, 3rd ed.; Morss, L. R.; Edelstein, N. M. & Fuger, J. Eds. Springer: Netherlands, **1**, 599-601 (2006).

Supplementary Table 7 | Refined parameters for fitted EXAFS data.

	POP-AO	POP-*p*NH_2-AO	POP-*o*NH_2-AO
S_o^2	1	1	1
ΔE_0 (eV)	0 ± 2	0 ± 3	0 ± 2
CN_O	7.2 ± 2.8	6.5 ± 2.6	6.4 ± 2.1
$CN_{C(CO3)}$	0.8 ± 0.6	1.0 ± 0.8	1.0 ± 0.6
$CN_{C(AO)}$	2.5 ± 1.2	2.3 ± 0.8	1.4 ± 1.1
Δr_{oyl} (Å)	0.013 ± 0.008	0.017 ± 0.009	0.014 ± 0.007
Δr_O (Å)	0.04 ± 0.02	0.04 ± 0.02	0.04 ± 0.02
$\Delta r_{C(CO3)}$ (Å)	0.02 ± 0.04	0.03 ± 0.05	0.02 ± 0.04
$\Delta r_{C(AO)}$ (Å)	-0.11 ± 0.03	-0.11 ± 0.04	-0.11 ± 0.05
σ^2_{oyl} ($\times 10^{-3}$ Å^2)	2.7 ± 0.4	2.6 ± 0.6	2.8 ± 0.4
σ^2_O ($\times 10^{-3}$ Å^2)	16 ± 5	13 ± 4	14 ± 4
σ^2_C ($\times 10^{-3}$ Å^2)	2.9 ± 0.1	3 ± 1	3.0 ± 0.7

Supplementary Note 1

The highest uranyl capture capacity from water given by POP-*p*NH_2-AO among the tested adsorbents can be reasonably attributed to the separate coordination between the uranyl-amidoxime and uranyl-amine in POP-*p*NH_2-AO. Interaction between the amino group and uranium is expected due to the successful uranyl extraction solely on an amine-based MOF (see reference 34). However, in the case of POP-*o*NH_2-AO, the amino group participates in the complex formation, serving as a reinforce group to enhance the coordinative interaction between amidoxime and uranyl, which does not bind with uranyl proven by the single crystal structure (see details below). However, in the presence of other competing ions, such as simulated seawater and seawater, sorbent material (POP-*o*NH_2-AO) with higher binding affinity towards uranium shows superior performance since the weak binding sites (amino group) may be unable to capture the target ions.

Supplementary Methods

Synthesis of porous adsorbent material constructed by benzamidoxime moieties (POP-AO).

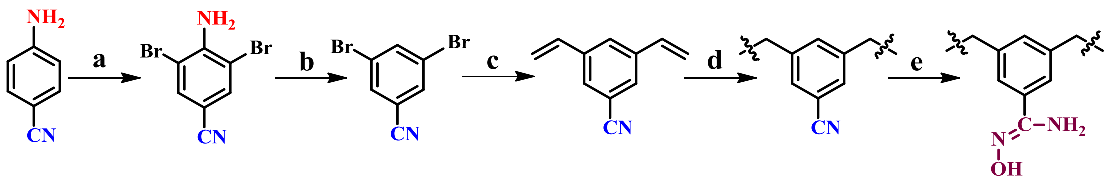

Reagents: (a) Br_2; (b) H_2SO_4, $NaNO_2$, Cu; (c) potassium vinyltrifluoroborate, $Pd(OAc)_2$; (d) AIBN; (e) $NH_2OH\cdot HCl$

4-amino-3,5-dibromobenzonitrile. To a solution of 4-aminobenzonitrile (6.0 g, 51 mmol) in acetic acid (100 mL), Br_2 (6.0 mL, 117 mmol) was added slowly. After being stirred at room temperature overnight, the mixture was poured into ice, and the crude bromo-derivative was filtered, washed with $NaHSO_3$ solution, dissolved in CH_2Cl_2, dried by K_2CO_3, and purified by flash chromatography with hexane/ethyl acetate (5:1) as eluent to give the title compound as a white solid. Yield: 13.2 g (95%). ^{1}H NMR (400 MHz, $CDCl_3$, 298K, TMS): δ 7.62 (s, 2H), 5.09 (s, 2H) ppm.

3,5-dibromobenzonitrile. Concentrated sulphuric acid (10 mL) was added gradually to a solution of 4-amino-3,5-dibromobenzonitrile (4.1 g, 15 mmol) in benzene (15 mL) and ethanol (100 mL) at room temperature, after which the reaction mixture was refluxed for 3 h. To the residue, a solution of sodium nitrite (2.8 g, 40 mmol) in a small amount of water was added gradually at 0 °C, followed by copper powder (0.96 g, 15 mmol). After being stirred at room temperature overnight and refluxed for a further 3 h, the insoluble materials were removed by filtration. The filtrate was extracted with ethyl acetate, washed with water, dried over Na_2SO_4, and evaporated under reduced pressure to give the crude compound which was purified by flash chromatography with hexane/ethyl acetate (20:1) as eluent to afford the title compound as a white solid. Yield: 3.4 g (87%). ^{1}H NMR (400 MHz, $CDCl_3$, 298K, TMS): δ 7.89 (t, 1H, *J*=1.4 Hz), 7.72 (t, 2H, *J*=0.8 Hz) ppm.

3,5-divinylbenzonitrile. 3,5-dibromobenzonitrile (2.0 g, 7.7 mmol), potassium vinyltrifluoroborate (2.06 g, 18.5 mmol), K_2CO_3 (4.25 g, 30.8 mmol), PPh_3 (0.08 g, 0.308 mmol), and $Pd(OAc)_2$ (0.034 g, 0.154 mmol) were dissolved in a mixture of toluene (25 mL), THF (25 m L), and H_2O (5 mL) and the resulting mixture was refluxed at 90 °C under N_2 atmosphere for 12 h. The residue was extracted with ethyl acetate, washed with brine, dried over Na_2SO_4, and evaporated under reduced pressure to give the crude compound which was purified by flash chromatography with hexane/ethyl acetate (20:1) as eluent to afford the title compound as a transparent liquid. Yield: 1.1 g (92%). ^{1}H NMR (500 MHz, $CDCl_3$, 298K, TMS): δ 7.89 (s, 1H), 7.55 (d, 2H, *J*=1.5 Hz),

6.66-6.72 (m, 2H), 5.83 (d, 2H, J=17.5 Hz), 5.41 (d, 2H, J=11.0 Hz) ppm. ^{13}C NMR (125 MHz, $CDCl_3$, 298K, TMS) 113.09, 116.80, 116.69, 128.20, 128.66, 134.77, 138.98.

Synthesis of porous polymer constructed by benzonitrile moieties (POP-CN). 3,5-divinylbenzonitrile (1.0 g) was dissolved in DMF (10 mL), followed by the addition of azobisisobutyronitrile (AIBN, 0.025 g). The mixture was transferred into a 20 mL autoclave and maintained for 24 h at 100 °C. A white solid product (1.0 g, 100% yield) was obtained after being washed with ethanol and dried under vacuum at 50 °C for 24 h.

Synthesis of porous adsorbent material constructed by benzamidoxime moieties (POP-AO). As a typical synthesis recipe, POP-CN (0.2 g) was swollen in ethanol (20 mL) for 10 min, followed by the addition of $NH_2OH\cdot HCl$ (0.5 g) and $N(CH_2CH_3)_3$ (0.75 g). After being stirred at 70 °C for 48 h to convert the nitrile into amidoxime, the mixture was filtered, washed with excess water, and finally dried at 50 °C under vacuum. The white solid obtained was denoted as POP-AO. POP-AO was treated with 3% (w/w) potassium hydroxide aqueous solution at room temperature for 36 h before adsorption tests.

Synthesis of porous adsorbent material constructed by 4-amino-benzamidoxime moieties (POP-pNH$_2$-AO).

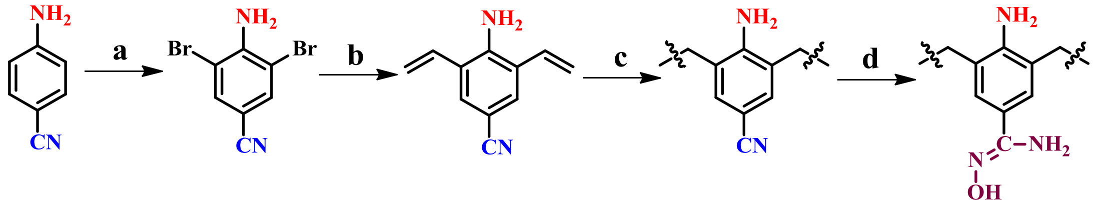

Reagents: (a) Br_2, AcOH; (b) potassium vinyltrifluoroborate, $Pd(OAc)_2$; (c) AIBN; (d) NH_2-OH·HCl

4-amino-3,5-dibromobenzonitrile. To a solution of 4-aminobenzonitrile (6.0 g, 51 mmol) in acetic acid (100 mL), Br_2 (6.0 mL, 117 mmol) was added slowly. After being stirred at room temperature overnight, the mixture was poured into ice, and the crude bromo-derivative was filtered, washed with $NaHSO_3$ solution, dissolved in CH_2Cl_2, dried by K_2CO_3, and purified by flash chromatography with hexane/ethyl acetate (5:1) as eluent to give the title compound as a white solid. Yield: 13.2 g (95%). ^{1}H NMR (400 MHz, $CDCl_3$, 298K, TMS): δ 7.62 (s, 2H), 5.09 (s, 2H) ppm.

4-amino-3,5-divinylbenzonitrile. 4-amino-3,5-dibromobenzonitrile (2.0 g, 7.2 mmol), potassium vinyltrifluoroborate (2.06 g, 18.5 mmol), K_2CO_3 (4.25 g, 30.8 mmol), PPh_3 (0.08 g, 0.308 mmol), and $Pd(OAc)_2$ (0.034 g, 0.154 mmol) were dissolved in a mixture of toluene (25 mL), THF (25 m L), and H_2O (5 mL) and the resulting mixture was refluxed at 90 °C under N_2 atmosphere for 12 h. The residue was extracted with ethyl acetate, washed with brine, dried over Na_2SO_4, and

evaporated under reduced pressure, giving the crude compound which was purified by flash chromatography with hexane/ethyl acetate (5:1) as eluent to afford the title compound as a yellow solid. Yield: 1.05 g (85%). ^{1}H NMR (500 MHz, $CDCl_3$, 298K, TMS): δ 7.43 (s, 2H), 6.64-6.69 (m, 2H), 5.66 (d, 2H, *J*=17.0 Hz), 5.70 (d, 2H, *J*=16.0 Hz), 5.46 (d, 2H, *J*=11.0 Hz) ppm. ^{13}C NMR (125 MHz, $CDCl_3$, 298K, TMS) 100.75, 119.08, 119.93, 124.7, 130.83, 131.17, 144.81.

Synthesis of porous polymer constructed with 4-amino-benzonitrile (POP-pNH$_2$-CN): 4-amino-3,5-divinylbenzonitrile (1.0 g) was dissolved in DMF (10 mL), followed by the addition of AIBN (25 mg). The mixture was transferred into a 20 mL autoclave and maintained for 24 h at 100 °C. A yellow solid product (1.0 g, 100% yield) was obtained after being washed with ethanol and dried under vacuum at 50 °C for 24 h.

Synthesis of porous adsorbent material constructed by 4-amino-benzamidoxime moieties (POP-pNH$_2$-AO). As a typical synthesis recipe, POP-*p*NH$_2$-CN (0.2 g) was swollen in ethanol (20 mL) for 10 min, followed by the addition of $NH_2OH·HCl$ (0.5 g) and $N(CH_2CH_3)_3$ (0.75 g). After being stirred at 70 °C for 48 h to convert the nitrile into amidoxime, the mixture was filtered, washed with excess water, and finally dried at 50 °C under vacuum. The light brown solid obtained was denoted as POP-*p*NH$_2$-AO. POP-*p*NH$_2$-AO was treated with 3% (w/w) potassium hydroxide aqueous solution at room temperature for 36 h before adsorption tests.

Synthesis of porous adsorbent material constructed by 2-amino-benzamidoxime moieties (POP-oNH$_2$-AO).

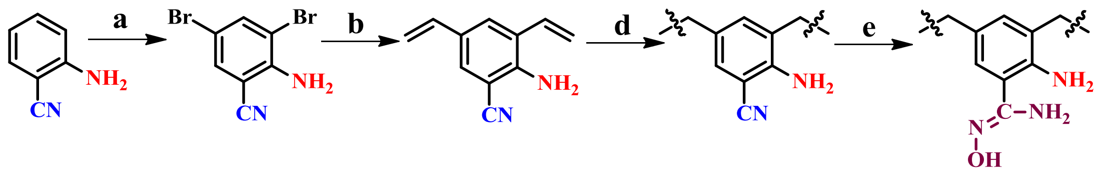

Reagents: (a) Br_2, AcOH; (b) potassium vinyltrifluoroborate, $Pd(OAc)_2$; (c) AIBN; (d) NH_2-OH·HCl

2-amino-3,5-dibromobenzonitrile. To a solution of 2-aminobenzonitrile (6.0 g, 51 mmol) in acetic acid (100 mL), Br_2 (6.0 mL, 117 mmol) was added slowly. After being stirred at room temperature overnight, the mixture was poured into ice, and the crude product was filtered, washed with $NaHSO_3$ solution, dissolved in CH_2Cl_2, dried by K_2CO_3, and purified by flash chromatography with hexane/ethyl acetate (5:1) as eluent to give the title compound as a light yellow solid. Yield: 12.8 g (92%). ^{1}H NMR (400 MHz, d_6-DMSO, 298K, TMS): δ 7.86 (d, 1H, *J*=2.4 Hz), 7.72 (d, 1H, *J*=2.4 Hz), 6.25 (s, 2H) ppm.

2-amino-3,5-divinylbenzonitrile. 2-amino-3,5-dibromobenzonitrile (2.0 g, 7.2 mmol), potassium vinyltrifluoroborate (2.06 g, 18.5 mmol), K_2CO_3 (4.25 g, 30.8 mmol), PPh_3 (0.08 g, 0.308 mmol), and $Pd(OAc)_2$ (0.034 g, 0.154 mmol) were dissolved in a mixture of toluene (25 mL), THF (25

mL), and H_2O (5 mL) and the resulting mixture was refluxed at 90 °C under N_2 atmosphere for 12 h. The residue was extracted with ethyl acetate, washed with brine, dried over Na_2SO_4, and evaporated under reduced pressure, giving the crude compound which was purified by flash chromatography with hexane/ethyl acetate (5:1) as eluent to afford the title compound as yellow solid. Yield: 0.95 g (77%). ^{1}H NMR (400 MHz, d_6-DMSO, 298K, TMS): δ 7.63 (d, 1H, *J*=1.6 Hz), 7.48 (d, 1H, *J*=2 Hz), 6.49-6.95 (m, 2H), 6.04 (s, 2H), 5.64-5.75 (m, 2H), 5.03-5.28 (m, 2H) ppm. ^{13}C NMR (100 MHz, d_6-DMSO, 298K, TMS): 148.22, 135.28, 131.62, 130.53, 128.61, 126.29, 123.74, 118.30, 116.59, 112.06, 95.64.

Synthesis of porous polymer constructed with 2-amino-benzonitrile (POP-oNH$_2$-CN). 2-amino-3,5-divinylbenzonitrile (1.0 g) was dissolved in DMF (10 mL), followed by the addition of AIBN (25 mg). The mixture was transferred into a 20 mL autoclave and maintained for 24 h at 100 °C. A yellow solid product (1.0 g, 100% yield) was obtained after being washed with ethanol and dried under vacuum at 50 °C for 24 h.

Synthesis of porous adsorbent material constructed by 2-amino-benzamidoxime moieties (POP-oNH$_2$-AO). As a typical synthesis recipe, POP-*o*NH$_2$-CN (0.2 g) was swollen in ethanol (20 mL) for 10 min, followed by the addition of NH_2OH·HCl (0.5 g) and $N(CH_2CH_3)_3$ (0.75 g). After being stirred at 70 °C for 48 h to convert the nitrile into amidoxime, the mixture was filtered, washed with excess water, and finally dried at 50 °C under vacuum. The light brown solid obtained was denoted as POP-*o*NH$_2$-AO. POP-*o*NH$_2$-AO was treated with 3% (w/w) potassium hydroxide aqueous solution at room temperature for 36 h before adsorption tests.

Sorption Experiments

The aqueous solutions with different uranium concentrations were obtained by diluting the stock $UO_2(NO_3)_2·6H_2O$ solution with the proper amount of distilled water unless otherwise indicated. The pH levels of the solutions were adjusted by HNO_3 or NaOH aqueous solution. The concentrations of uranium during all the experiments were detected by inductively coupled plasma-optical emission spectroscopy (ICP-OES) and inductively coupled plasma-mass spectrometry (ICP-MS) for extra low concentrations. All the adsorption experiments were performed at ambient conditions.

Uranium sorption isotherms. To obtain the uranium adsorption isotherms for various adsorbents, POP-AO (5 mg), POP-*p*NH$_2$-AO (5 mg), or POP-*o*NH$_2$-AO (5 mg) was added into 10 mL aqueous solutions with different concentrations of uranium in the range of 36 to 356 ppm. Adsorbents were suspended fully by a brief sonication and then the mixtures were stirred vigorously overnight, by which time it was assumed that adsorption equilibrium had been reached. The treated solutions were filtrated through a 0.45-um membrane filter. The supernatant was analyzed using ICP analysis to determine the remaining uranium concentration. The adsorbed amount at equilibrium (q_e, mg

g^{-1}) was calculated by:

$$q_e = \frac{(C_i - C_e) \times V}{m}$$

where V is the volume of the treated solution (mL) and m is the amount of adsorbent used (g), and C_i and C_e are the initial concentration and the final equilibrium concentration of uranium, respectively.

Uranium sorption kinetics from distilled water. Uranium aqueous solution (400 mL, 7.56 ppm) and adsorbents (3 mg) were added to an Erlenmeyer flask with a magnetic stir bar. The mixture was stirred at room temperature for 3 h. At appropriate time intervals, aliquots (5 mL) were taken from the mixture, and the adsorbents were separated by syringe filter (0.45 μm membrane filter). The uranium concentrations in the resulting solutions were analyzed by ICP-OES. The adsorption capacity at different intervals was calculated as follows:

$$\text{Adsorption capacity (mg/g)} = (C_i - C_t) \times V/m$$

where V is the volume of the treated solution (mL) and m is the amount of used adsorbent (mg), and C_i and C_t are the initial concentration and the concentration of uranium at t (min), respectively.

Uranium uptake capacities from distilled water with various pH values. Uranium aqueous solution (400 mL, 7.56 ppm) and adsorbents (3 mg) were added to an Erlenmeyer flask with a magnetic stir bar. After being stirred at room temperature for 3 h, aliquots were taken from the mixture, and the adsorbents were separated by syringe filter (0.45 μm membrane filter). The uranium concentrations in the resulting solutions were analyzed by ICP-OES and the uptake capacities were calculated based on the aforementioned equations.

Uranium removal kinetics from distilled water. Uranium aqueous solution (250 mL, 3560 ppb) and adsorbents (5 mg) were added to an Erlenmeyer flask with a magnetic stir bar. The mixture was stirred at room temperature for 1 h. At appropriate time intervals, aliquots (5 mL) were taken from the mixture, and the adsorbents were separated by syringe filter (0.45 μm membrane filter). The uranium concentrations in the resulting solutions were analyzed by ICP-MS. The percentage removal of UO_2^{2+} was calculated as follows:

$$\text{Removal percentage (\%)} = \frac{C_i - C_t}{C_0} \times 100$$

Uranium removal kinetics from potable water. Potable water spiked with 1000 ppb uranium (250 mL) and adsorbents (5 mg) were added to an Erlenmeyer flask with a magnetic stir bar. The mixture was stirred at room temperature for 1 h. At appropriate time intervals, aliquots (5 mL) were taken from the mixture, and the adsorbents were separated by syringe filter (0.45 μm membrane filter). The uranium concentrations in the resulting solutions were analyzed by ICP-MS.

Uranium removal efficiency from potable water at various phase ratios. Potable water spiked with 1000 ppb uranium and adsorbents at various phase ratios listed in Supplementary Figure 12 were introduced into an Erlenmeyer flask with a magnetic stir bar. After being stirred at room temperature for 3 h, aliquots were taken from the mixture, and the adsorbents were separated by syringe filter (0.45 μm membrane filter). The uranium concentrations in the resulting solutions were analyzed by ICP-MS.

Selectivity tests. To a 200 mL solution of potable water spiked with UO_2^{2+}, Cu^{2+}, Fe^{3+}, Co^{2+}, Pb^{2+}, Zn^{2+}, La^{3+}, Ce^{3+}, Sm^{3+}, Cs^{+}, Sr^{2+}, Mg^{2+}, and Ca^{2+} at a concentration of *ca.* 1000 ppb in an Erlenmeyer flask with a magnetic stir bar, POP-*o*NH_2-AO (5 mg) was added. After being stirred at room temperature for 1 h, aliquots were taken from the mixture, and the adsorbents were separated by syringe filter (0.45 μm membrane filter). The uranium concentration in the resulting solutions were analyzed by ICP-MS. To evaluate the removal efficiency of POP-*o*NH_2-AO (5 mg) towards uranium species in the presence of a large excess of aforementioned ions, tests were performed using a potable water sample (100 mL) containing uranium (*ca.* 1 ppm) and the ions aforementioned with nearly equal concentrations (*ca.* 500 ppm). After being stirred at room temperature for 1 h, aliquots were taken from the mixture, and the adsorbents were separated by syringe filter (0.45 μm membrane filter). The uranium concentration in the resulting solutions were analyzed by ICP-OES.

Uranium sorption kinetics from simulated seawater. Simulated seawater (25.6 g L^{-1} NaCl and 0.198 g L^{-1} $NaHCO_3$) spiked with 10.3 ppm uranium (400 mL) and adsorbents (3 mg) were added to an Erlenmeyer flask with a magnetic stir bar. The mixture was stirred at room temperature for 300 min. At appropriate time intervals, aliquots (5 mL) were taken from the mixture, and the adsorbents were separated by syringe filter (0.45 μm membrane filter). The uranium concentrations in the resulting solutions were analyzed by ICP-OES.

Uranium removal kinetics from simulated seawater. Simulated seawater (25.6 g L^{-1} NaCl and 0.198 g L^{-1} $NaHCO_3$) spiked with 4056 ppb uranium (10 mL) and adsorbents (5 mg) were added to an Erlenmeyer flask with a magnetic stir bar. The mixture was stirred at room temperature. At appropriate time intervals, aliquots were taken from the mixture, and the adsorbents were separated by syringe filter (0.45 μm membrane filter). The uranium concentrations in the resulting solutions were analyzed by ICP-MS.

Uranium extraction from uranium spiked seawater. Seawater spiked with 10.3 ppm uranium (400 mL) and adsorbents (3 mg) were added to an Erlenmeyer flask with a magnetic stir bar. After being stirred at room temperature for 24 h, aliquots were taken from the mixture, and the adsorbents were separated by syringe filter (0.45 μm membrane filter). The uranium concentrations in the resulting solutions were analyzed by ICP-OES.

Uranium enrichment from real seawater. Adsorbent material (5 mg) was immersed in a tank

containing 5 gallons of seawater and shaken at 100 rpm at room temperature. After 56 days, the adsorbent was collected by filtration, washed with water, and dried at 80 °C under vacuum for 24 h. The amount of uranium enriched in the adsorbent was determined by ICP-MS analysis after being digested by aqua regia.

X-ray Crystallography

The X-ray diffraction data was measured on Bruker D8 Venture PHOTON 100 CMOS system equipped with a Cu K_{α} INCOATEC ImuS micro-focus source (λ = 1.54178 Å). Indexing was performed using *APEX3*[1] (Difference Vectors method). Data integration and reduction were performed using SaintPlus 6.01[2]. Absorption correction was performed by multi-scan method implemented in SADABS[3]. Space groups were determined using XPREP implemented in APEX3[1]. Structures were solved using SHELXT and refined using SHELXL-2016[3-7] (full-matrix least-squares on F^2) through OLEX2 interface program[8]. All non-hydrogen atoms were refined anisotropically. Hydrogen atoms of -CH and $-CH_3$ groups were placed in geometrically calculated positions and were included in the refinement process using riding model with isotropic thermal parameters: Uiso(H) = 1.2(1.5)Ueq(-CH, ($-CH_3$)). Hydrogen atoms of -OH and $-NH_2$ groups were refined with DFIX/DANG or using riding model. Pseudo translational effects present in the data are due to the presence and perfect alignment of heavy uranium cations in c direction. Structure solution based on data integrated with halved c unit cell parameter resulted however with heavy disorder of uranium chelating ligand and O atoms. Crystal data and refinement conditions are shown in Supplementary Table 2.

Computational Methods

Electronic structure calculations. Quantum chemical calculations were performed with the Gaussian 09 D.01 software[9]. We adopted the density functional theory (DFT) approach for our calculations using the M06[10] density functional with the standard Stuttgart small-core (SSC) 1997 relativistic effective core potential (RECP)[11] and the associated contracted [8s/7p/6d/4f] basis set for uranium atom, along with the 6-311++G(d,p) basis set for the light atoms. Frequency calculations were performed at the B3LYP/SSC/6-31+G(d)[12] level to ensure that geometries (optimized at the same B3LYP/SSC/6-31+G(d) level) were minima and to compute zero-point energies and thermal corrections. Using the gas-phase geometries, implicit solvent corrections were obtained at 298 K with the SMD[13] solvation model as implemented in Gaussian 09 at the B3LYP/SSC/6-31+G(d) level of theory. The results are reported using the lowest energy clusters identified at the M06/SSC/6-311++G(d,p) level for a given stoichiometry and binding motif. The preference for using a combination of the M06 and the B3LYP functionals with the SMD solvation model was based on the results of our previous studies[14,15], which showed that the chosen level of theory provides the best overall performance in predicting the log *ß* values of uranyl complexes

with anionic oxygen and amidoxime donor ligands.

Ligand-UO_2^{2+} interactions. Assessment of second-order stabilization energies ($E^{(2)}$, kcal/mol) in $UO_2(AO)_2$, $UO_2(p\text{NH}_2\text{-AO})_2$, and $UO_2(o\text{NH}_2\text{-AO})_2$ complexes was performed with the natural bond orbital (NBO) method[16] at M06/SSC/6-311++G(d,p) using commercial stand-alone NBO 6.0 program[17]. It is worth noting that total electron densities derived from effective core potential (ECP) calculations may lead to artifacts in the topological analysis[18], however, NBO derived properties appear to be less critical in this respect and showed a remarkable consistency between ECP and scalar relativistic all-electron calculation schemes[19], justifying our choice towards ECP for the description of chemical bonding. The donor-acceptor interaction energy (second-order stabilization energies ($E^{(2)}$) in the NBOs was estimated via second-order perturbation theory analysis of the Fock matrix[16]. For each donor orbital (i) and acceptor orbital (j), the stabilization energy $E^{(2)}$ associated with $i \rightarrow j$ delocalization is given by:

$$E_{i,j}^{(2)} = -o_i \frac{\langle i | \hat{F}_{(i,j)} | j \rangle^2}{\varepsilon_j - \varepsilon_i}$$

, where o_i is the donor orbital occupancy, $\hat{F}_{(i,j)}$ is the Fock operator, and ε_i and ε_j are the orbital energies.

Calculations of pK_a, complexation free energies and stability constants. For the pK_a predictions we have adopted a protocol described in Supplementary ref20 which showed the best overall performance for a set of 13 oxygen donor and 3 amidoxime-based ligands, with a RMSD of 0.46 pK_a. This methodology[20] involves MP2/aug-cc-pVTZ//MP2/aug-cc-pVDZ calculations with the SMD[13] solvation model.

Complexation free energies in aqueous solution, ΔG_{aq}, and stability constants, log β, were calculated using the methodology described in our previous work on UO_2^{2+} complexes[14,15]. According to the thermodynamic cycle shown in Supplementary Scheme 1, ΔG_{aq} is given by:

$$\Delta G_{aq} = \Delta G^{o}_{g} + \Delta\Delta G^{*}_{solv} + (n\text{-}1)\Delta G^{o\rightarrow *} + nRT\ln([H_2O])$$

where ΔG^{o}_{g} is the free energy of complexation in the gas phase and $\Delta\Delta G^{*}_{solv}$ is the difference in the solvation free energies for a complexation reaction:

$$\Delta\Delta G^{*}_{solv} = \Delta G^{*}_{solv}([ML(H_2O)_{m-n}]^{x+y}) + n\Delta G^{*}_{solv}(H_2O) - \Delta G^{*}_{solv}([M(H_2O)_m]^{x}) - \Delta G^{*}_{solv}(L^{y})$$

where L^y denotes the ligand with a charge of y and M is UO_2^{2+}. The standard state correction terms must be introduced to connect ΔG^{o}_{g}, $\Delta\Delta G^{*}_{solv}$, and ΔG_{aq}, which are defined using different standard state conventions. The free energy change for the conversion of 1 mol of solute from the gas phase at a standard state of 1 atm (24.46 L mol^{-1}) to the aqueous phase at a standard state of 1 mol L^{-1} at 298.15 K is given by $\Delta G^{o\rightarrow *}$ = 1.89 kcal mol^{-1}. Likewise, $RT\ln([H_2O])$ = 2.38 kcal mol^{-1} (T = 298.15 K) is the free energy change for the conversion of 1 mol of solvent from the aqueous phase at 1 mol L^{-1} to pure water at a standard state of 55.34 mol L^{-1}. Lastly, the stability constant (log β) is related to free energy change for the complexation reaction by the following equation:

$$\log\beta = \frac{-\Delta Gaq}{2.303 \cdot RT}$$

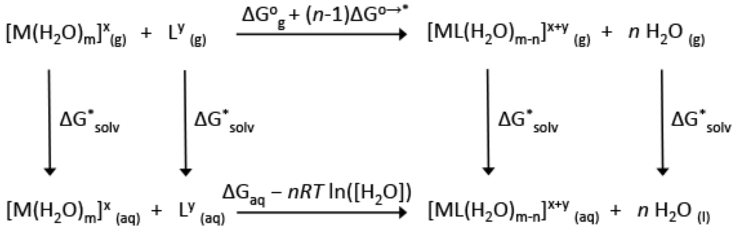

Supplementary Scheme 1 | Thermodynamic cycle used to calculate ΔG_{aq}.

Speciation diagrams were calculated using the HySS[21] program and formation constants for uranyl-carbonate complexes and uranyl hydroxy species are given in supplementary Table 6.

EXAFS

Sample Preparation. Approximately 20-25 mg of sample was enclosed within a nylon washer of 4.953 mm inner diameter (area of 0.193 cm^2), sealed on one side with transparent "Scotch" tape. The sample was pressed thoroughly by hand to form a firm, uniform pellet, and then sealed on the open side with a second piece of tape. The entire sample was placed into a Mylar baggie. Small pieces of Kapton tape were used to seal the three open edges of the Kapton baggie. This method was approved in advance by the APS Radiation Safety Review Board for achieving the double containment necessary for analysis of radioactive samples.

Data Collection. The X-ray absorption data were collected at Beamline 10BM-B at the Advanced Photon Source (APS) at Argonne National Laboratory. Spectra were collected at the uranium L_3-edge (17166 eV) in transmission mode. The X-ray white beam was monochromatized by a Si(111) monochromator and detuned by 50% to reduce the contribution of higher-order harmonics to below the level of noise. The K-edge of an yttrium foil (17038 eV) was used as the reference for energy calibration and measured simultaneously for all samples. The incident beam intensity (I_0), transmitted beam intensity (I_t), and reference (I_r) were all measured by 20 cm ionization chambers with gas compositions of 80% N_2 and 20% Ar, 95% Ar and 5% N_2, and 100% N_2, respectively. All spectra were collected at room temperature.

Samples were centered on the beam and adjusted to find the most homogeneous location in the sample for data collection. The beam was reduced to dimensions of 400 × 3100 μm for all data collection. Data were collected over six regions: -250 to -30 eV (10 eV step size, dwell time of 0.25 seconds), -30 to -5 eV (5 eV step size, dwell time of 0.5 seconds), -5 to 30 eV (1 eV step size), 3 $Å^{-1}$ to 6 $Å^{-1}$ (0.05 $Å^{-1}$ step size, dwell time of 2 seconds), 6 $Å^{-1}$ to 9 $Å^{-1}$ (0.05 $Å^{-1}$ step size, dwell time of 4 seconds), and 9 $Å^{-1}$ to 15 $Å^{-1}$ (0.05 $Å^{-1}$ step size, dwell time of 8 seconds). Three scans were collected for each sample.

The data were processed and analyzed using the Athena and Artemis programs of the IFEFFIT package based on FEFF 6[22,23]. Reference foil data were aligned to the first zero-crossing of the

second derivative of the normalized μ(E) data, which was subsequently calibrated to the literature E_0 for the yttrium K-edge (17038 eV). Spectra were averaged in μ(E) prior to normalization. The background was removed and the data were assigned an Rbkg value of 1.0.

Fitting. All data were initially fit with k-weighting of 1, 2, and 3, then finalized with k^3-weighting in R-space. Structural parameters that were determined by the fits include the degeneracy of the scattering path (N_{degen}), the change in half-path length, R_{eff} (ΔR_i), the relative mean square displacement of the scattering element (σ^2_i), the passive electron reduction factor (S_0^2), and the energy shift of the photoelectron, (ΔE_0). S_0^2 was found to converge to 1.0 ± 0.10 for all fits and was thus fixed at that value for all models. For each fit, the number of independent points was not permitted to exceed 2/3 the number of variables, in keeping with the Nyquist criterion[24,25].

Fits of the data were attempted in a bottom-up fashion using the structure models as displayed in Fig. 5 of the main text. Fitting atomic degeneracy was achieved through the introduction of a variable which scaled the amplitude reduction factor, S_0^2. While more distant scattering paths were progressively included, refined values for previously established scattering paths were used as initial guesses, but allowed to vary freely to avoid introduction of systematic error. The final model used for all fits contained direct scattering paths off two axial oxygen, a variable number of light scatterers in the equatorial plane (O and N backscatterers are indistinguishable by EXAFS), a variable number of carbon scatterers at 2.89 Å representative of carbonate, and a variable number of carbon scatterers at 3.57 Å representative of amidoxime ligands bound in an η^2 configuration. A summary of the refined fit parameters for all systems is displayed below in supplementary Table 7.

Supplementary References

1. Bruker. *APEX3* (Version 2015.9). Bruker AXS Inc., Madison, Wisconsin, USA (2016).
2. Bruker SAINT V8.35A. Data Reduction Software (2016).
3. Sheldrick, G. M. *SADABS. Program for Empirical Absorption Correction.* University of Gottingen, Germany (1996).
4. Sheldrick, G. M. "Crystal structure refinement with SHELXL", Acta Cryst. C71, 3-8 (2015).
5. Sheldrick, G. M. Acta Cryst. A46, 467-473 (1990).
6. Sheldrick, G. M. Acta Cryst. A64, 112-122 (2008).
7. Sheldrick, G. M. Acta Cryst. A71, 3-8 (2015).
8. Dolomanov, O. V.; Bourhis, L. J.; Gildea, R. J.; Howard, J. A. K. & Puschmann, H. OLEX2: A complete structure solution, refinement and analysis program. *J. Appl. Cryst.* **42**, 339-341 (2009).
9. Frisch, M. J. et al. Gaussian, Inc. Wallingford CT (2009).
10. Zhao, Y. & Truhlar, D. G. The M06 suite of density functionals for main group thermochemistry, thermochemical kinetics, noncovalent interactions, excited states, and transition elements: two new functionals and systematic testing of four M06-class functionals and 12 other functionals. *Theor. Chem. Acc.* **120**, 215-241 (2008).
11. Dolg, M.; Stoll, H.; Preuss, H. & Pitzer, R. M. Relativistic and correlation effects for element 105 (Hahnium, Ha). A comparative study of M and MO (M= Nb, Ta, Ha) using energy-adjusted ab initio pseudopotentials. *J. Phys. Chem*. **97**, 5852 (1993).
12. Becke, A. D. Density-functional thermochemistry. III. The role of exact exchange. *J. Chem. Phys.* **98**, 5648-565 (1993).
13. Marenich, A. V.; Cramer, C. J. & Truhlar, D. G. Universal solvation model based on solute electron density and on a continuum model of the solvent defined by the bulk dielectric constant and atomic surface tensions. *J. Phys. Chem. B* **113**, 6378-6396 (2009).
14. Vukovic, S.; Hay, B. P. & Bryantsev, V. S. Predicting stability constants for uranyl complexes using density functional theory. *Inorg. Chem*. **54**, 3995-4001 (2015).
15. Mehio, N.; Ivanov, A. S.; Williams, N. J.; Mayes, R. T.; Bryantsev, V. S.; Hancock, R. D. & Dai, S. Quantifying the binding strength of salicylaldoxime-uranyl complexes relative to competing salicylaldoxime-transition metal ion complexes in aqueous solution: a combined experimental and computational study. *Dalton. Trans.* **45**, 9051-9064 (2016).
16. Foster, J. P. & Weinhold, F. "Natural hybrid orbitals." *J. Am. Chem. Soc.* **102**, 7211-7218 (1980).
17. Glendening, E. D.; Landis, C. R. & Weinhold, F. "NBO 6.0: natural bond orbital analysis program." *J. Comput. Chem*. **34**, 1429-1437 (2013).
18. Vyboishchikov, S. F.; Sierraalta, A. & Frenking, G. J. Topological analysis of electron density distribution taken from a pseudopotential calculation. *Comput. Chem*. **18**, 416-429 (1997).
19. Pantazis, D. A.; Chen, X.-Y.; Landis, C. R. & Neese, F. All-electron scalar relativistic basis sets for third-row transition metal atoms. *J. Chem. Theory Comput.* **4**, 908-919 (2008).
20. Mehio, N.; Lashely, M. A.; Nugent, J. W.; Tucker, L.; Correia, B.; Do-Thanh, C.-L.; Dai, S.; Hancock, R. D. & Bryantsev, V. S. Acidity of the amidoxime functional group in aqueous solution: a combined experimental and computational study. *J. Phys. Chem. B* **119**, 3567-3576 (2015).

21. Alderighi, L.; Gans, P.; Ienco, A.; Peters, D.; Sabatini, A. & Vacca, A. Hyperquad simulation and speciation (HySS): a utility program for the investigation of equilibria involving soluble and partially soluble species. *Coord. Chem. Rev.* **184**, 311-318 (1999).
22. Ravel, B. & Newville, M. Athena, artemis, hephaestus: data analysis for X-ray absorption spectroscopy using IFEFFIT. *J. Synchrotron Radiat.* **12**, 537-541 (2005).
23. Rehr, J. J. & Albers, R. C. Theoretical approaches to X-ray absorption fine structure. *Rev. Mod. Phys.* **72**, 621-654 (2000).
24. Calvin, S. *XAFS for Everyone*. CRC Press: Boca Raton, FL (2013).
25. Kelly, S. D.; Kemner, K. M.; Fein, J. B.; Fowle, D. A.; Boyanov, M. I.; Bunker, B. A. & Yee, N. X-ray absorption fine structure determination of pH-dependent U-bacterial cell wall interactions. *Geochim. Cosmochim. Acta* **66**, 3855-3871 (2002).
